# Pre-Roman improvements to agricultural production: Evidence from livestock husbandry in late prehistoric Italy

**DOI:** 10.1371/journal.pone.0208109

**Published:** 2018-12-31

**Authors:** Angela Trentacoste, Ariadna Nieto-Espinet, Silvia Valenzuela-Lamas

**Affiliations:** 1 Institute of Archaeology, University of Oxford, Oxford, United Kingdom; 2 Consejo Superior de Investigaciones Científicas (CISC), Institució Milà i Fontanals, Archaeology of Social Dynamics, Barcelona, Spain; University at Buffalo - The State University of New York, UNITED STATES

## Abstract

Domestication of wild cattle, sheep, and pigs began a process of body size diminution. In most of Western Europe this process continued across prehistory and was not reversed until the Roman period. However, in Italy, an increase in livestock body size occurred during the Iron Age, earlier than the Western provinces. In order to better understand the nature and timing of this early increase in animal size, this paper presents a detailed regional study of taxonomic abundance and biometric data from zooarchaeological assemblages recovered from the Po and Venetian–Friulian Plains in northern Italy. Our results demonstrate a high level of regionality in the choice of species exploited, with husbandry systems focused on different domesticates, as well as regional differences in animal size. However, despite significant variation in species frequencies, settlement structure, and epigraphic tradition, all areas with sufficient data demonstrate similar significant changes in livestock body size. Cattle and sheep increased incrementally in size prior to the Roman conquest in all regions considered; surprisingly, pigs continued to decrease in size throughout later prehistory. The incremental pace and pan-regional character of the size change in cattle and sheep suggests an internally motivated phenomenon rather than herd replacement with a new larger population, as might follow colonisation or conquest. The divergence in size trends for bovids and suids suggests a noteworthy change in cattle and sheep herding practices during the Iron Age or final centuries of the Bronze Age, in contrast with greater continuity in pig management. Our analysis provides a thorough zooarchaeological synthesis for northern Italy and, for the first time, demonstrates that both cattle and sheep increased in size outside of Roman territory well before the conquest of this area. This study offers a basis for future chemical analyses (DNA, isotopes), which will further investigate the cause(s) of livestock size changes in northern Italy.

## Introduction

A reduction in body size is widely recognised consequence of animal domestication [1–3]. Although the initial timing of morphological changes and of the selective aims that led to their emergence are still matters of debate [4, 5], this decrease in the livestock size proved to be a persistent and progressive trend. Across Western Europe, domestic animals, and particularly cattle, continued to decrease in size between the Neolithic and Iron Age [6–13]. Various explanations for this phenomenon have been put forward, including preference for smaller more manageable animals [14], and intensification in herding strategies through sub-adult breeding [15]. Regardless of the origin of this size decrease, the Roman Empire had a significant impact on its trajectory.

Roman conquest brought about an end to the Iron Age and significant changes to the social and economic organisation of Western Europe [16–18], including to animal farming strategies [12]. New methods of production are evident in changes to the species exploited and–breaking with millennia of progressive size diminution–a significant increase in the size of livestock. Although changes were not uniform [19] and exceptions occur [20–21], an increase in animal size is visible across conquered territories: France [22–24], Belgium [25], the Netherlands [26], Germany [27, 28], Switzerland [29–31], Britain [32], and Spain [12, 33]. This transformation of livestock husbandry is thought to result from the introduction of different forms of animal production [21, 34].

Uniquely in Western Europe, significant increases in animal size and changes to livestock frequencies pre-date the Roman conquest of northern and central Italy by centuries [7, 35, 36]. High percentages of pig remains and growth in animal body size are apparent in Etruscan cities as well as Rome and its environs during the first millennium BC [35, 37–39]. These trends continued to intensify during the Imperial period [40], by which point they had become a hallmark of ‘Romanisation’ in other parts of the Empire [19]. Within north/central Italy, these characteristic developments are linked to the rise of urban Etruscan and Roman culture, as marginal areas of Italy do not consistently demonstrate similar changes [20, 35, 41]. Urbanism is argued to be a primary force in catalysing changes to species frequencies, due to the functional challenges of provisioning cities with protein [37, 42, 43] and the socio-economic benefits of producing surplus animals [35]. Thus, the ‘Roman’ high-pig pattern became established during a period of increasing urbanisation during the first millennium BC, but do changes in animal size have the same origin?

Compared to species representation, the evolution of animal size in late prehistoric Italy is less understood. Because livestock varied significantly in stature across the Italian peninsula [7, 41, 42, 44, 45], analyses must be conducted on a sub-regional scale in order to establish useful patterns; a larger frame of analysis will convolute diachronic trends because the regional distribution of sites is not constant through time. A few late Bronze/early Iron Age sites have produced livestock with withers’ heights taller than those recorded in for earlier phases [45], but without detailed understanding of regional patterns of size evolution, the significance of these examples remains unclear: do they represent the origin of an accelerating trend towards larger animals that culminates in the Roman period? Or do they only appear large because comparative data are drawn from a different geographic area with smaller animals? In order to establish the origin and potential catalysts for animal size change, higher-resolution understanding of its development is needed.

### Aims

This paper examines frequencies of domestic animals and development of livestock size within northern Italy in order to better understand the evolution of animal husbandry strategies and the socio-economic and ecological factors that impacted it. Northern Italy provides an ideal setting to pursue this line of inquiry. The region demonstrates significant and well documented changes in husbandry regimes across later prehistory [45–50], including the emergence of the recognised ‘urban Etruscan/Roman’ trend for larger animals and abundant pigs [7, 35, 51, 52]. These developments took place in an interconnected and climatically comparable geographic context. The Po Valley, Italy’s largest alluvial basin, joins the adjacent Venetian–Friulian Plain to form a unified landscape across most of the region. Within this area, local conditions would have varied dependant on land use, forest/vegetation cover, and fluvial networks, but the territory is subject to a similar climate (Köppen–Geiger climate zone Cfa), albeit with slightly cooler temperatures and greater precipitation on the higher edges of the valley (Cfb) [53]. Limiting the study area to a well-connected region with comparable climatic constraints allows for better assessment of the role of socio-economic and cultural factors in shaping husbandry strategies.

**Fig 1:**
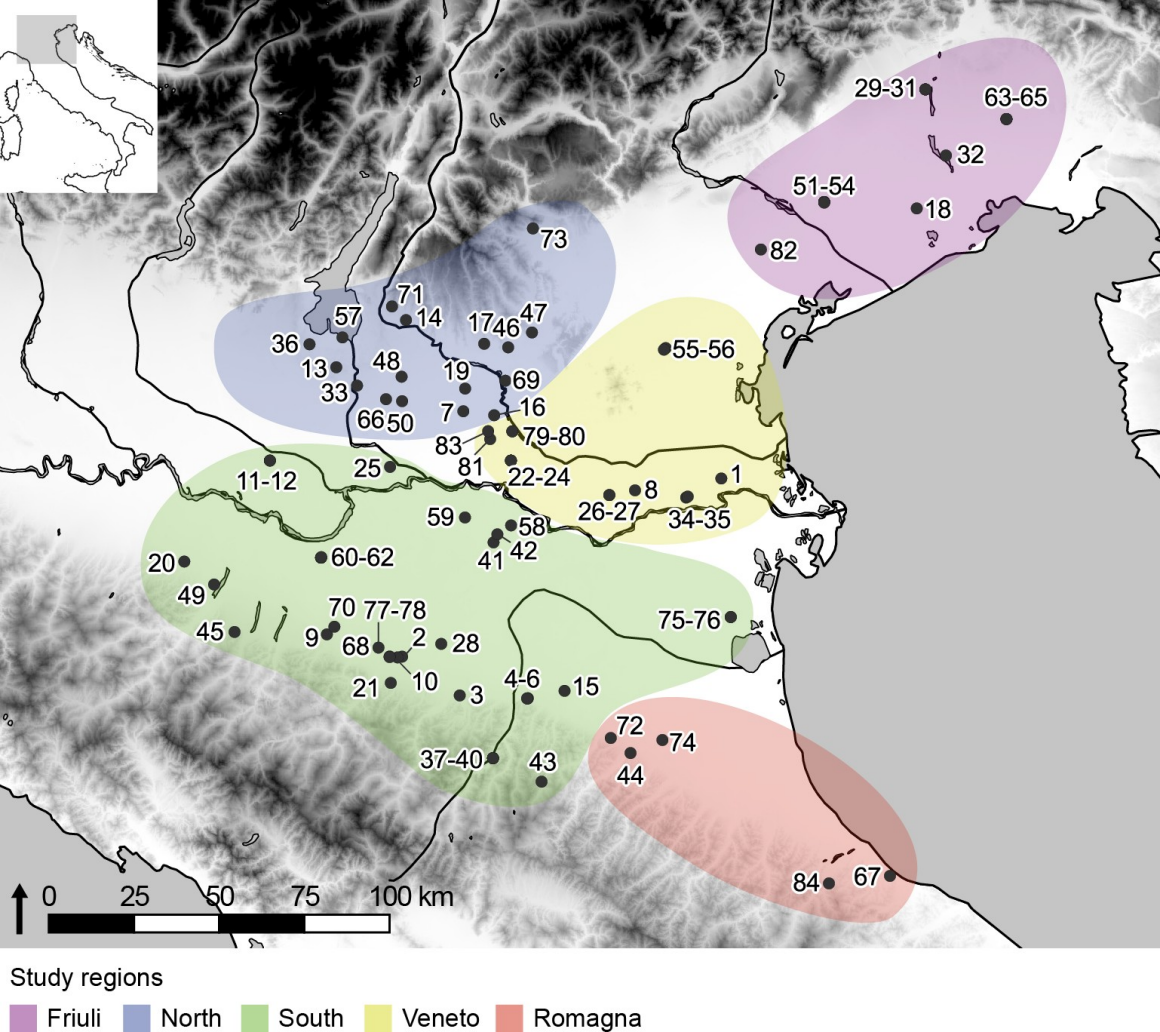
Map of sites and study regions. See S1 Table for details. Terrain data available from the U.S. Geological Survey.

Within this region, we compare five study areas (Fig 1) with distinct archaeological trajectories. In anticipation of subsequent research on the Roman transition, here we focus on later prehistory, specifically the Middle Bronze Age to late Iron Age, c. 1650–150 BC [54–57]. While the process was not linear or continuous [56], this time frame encompasses a period of increasing social complexity and regionalisation, and the transition from diffuse villages to cities in the southern and eastern Po Valley [54, 57]. Of particular significance to this study is the development of urban Etruscan culture in the South study area [58].

We aim to address three questions: How does livestock representation change through time? When/where do larger animals emerge? And how do changes in species representation relate to developments in animal size? These analyses will provide new data on the geographic distribution of husbandry regimes and on the nature of diachronic change (e.g. rapid/progressive). This evidence allows us to establish whether Etruscan/Roman animal-exploitation patterns (abundant pigs, large animals) are indeed unique to these cultures, and to what extent urban settlement structures were perquisite for improvements in livestock. In light of current evidence for animal management regimes particular to urban Etruscan and Roman culture [7, 35, 39, 51, 52], we hypothesise that changes in species representation and growth in animal size reflect a new agricultural strategy related to urban settlement networks, and anticipate greater continuity in other, non-urban parts of the study area.

## Archaeological context

Broadly considered, later prehistory saw demographic growth alongside population consolidation, social hierarchisation, and an intensification of production/trade, although these phenomena were neither linear nor homogenous.

The Early Bronze Age (c. 2300–1700 BC) of the Po Valley was characterised by pile-dwelling ‘palafitte’ villages, which represented part of a larger phenomenon of lake-side settlement in Switzerland and Central-Eastern Europe more generally [59]. During the Middle Bronze Age (c. 1700–1350 BC) the number of palafitte villages near lake basins declined, while a new form of settlement–terramare–spread along fluvial courses of the Po Valley [56, 59, 60]. Terramare villages used structural elements similar to palafitte, but were enclosed by moat and earthen rampart that functioned as part of the site’s irrigation network, as well as serving a defensive function [61]. Generally 1–2 ha in size, terramare displayed a high degree of internal organisation. Their dense distribution and use of complex irrigation and water management systems points significant population growth [56] and an intense exploitation of the territory [62, 63]. Significant tracks of the region’s forests were cleared, especially in the southern Po Plain, to supply building material and create space for agriculture and grazing [63–69]. Friuli also developed a more complex settlement pattern, but comparable with Istria, with fortified *castellieri* sites on hills [70].

Agriculture was based on cultivation of cereals and, to a lesser extent, legumes [63, 71, 72]. Millets were introduced as cultivars [73, 74]. This diversification of crops allowed the possibility of more than one annual harvest. Ploughs were used to prepare soil for planting, and material evidence for wooden ploughs in northern Italy dates back to Early Bronze Age [75]. Crop rotation, manuring, and fallowing were probably used to maintain or improve field productivity [76, 77]. The organisation of fields is difficult to reconstruct, but archaeobotanical evidence suggests a division of land between garden plots and extensive fields for cereals [77]; a similar land-use strategy has been proposed for Bronze Age agriculture in Switzerland [78, 79]. The diffusion of metal agricultural tools improved farming practices [75, 80], and the expansion of metallurgy may also have encouraged seasonal exploitation of upland pastures near metal sources [81]. Animals kept within settlements during cold months were foddered with twigs, grasses, and herbs [82].

The Recent Bronze Age (c. 1350–1150 BC) saw further changes in settlement patterns. Some sites were abandoned, while others increase in size; certain terramare villages reached an area of 15–20 ha [56, 59]. The development of organised settlements on this scale, alongside greater specialisation in metal production, and the distribution of status items in necropoli suggest more hierarchic forms of social organisation than visible in previous centuries [83, 84]. At the end of the Recent Bronze Age terramare-palafitte culture collapsed. Settlements were abandoned, leading to an almost complete depopulation of part of the southern Po Plain [56]. Aridification of environments already subject to deforestation and intensive cultivation appear to have had a significant role [62, 63, 85]; movement towards a more hierarchal society also may have caused a social crisis, exacerbated by environmental degradation [56]. While a large part of the southern plain was depopulated, the lowlands southeast of Verona and northern hills display greater settlement continuity [86, 87], and during the subsequent Final Bronze Age this area developed into the economic fulcrum of Po Valley. Settlement density in the Friulian lowlands increased before sharply falling [88].

The Final Bronze Age (c. 1150–950 BC) saw population consolidation along rivers and communication routes. Settlements developed into the first large clusters with proto-urban characteristics, the most important of which was Frattesina [89], which demonstrated a high level of complexity in craft production with the import of raw materials from as far as Egypt and the Baltic [90]. Shared forms of material culture and funerary practice (cremation) associated with proto-Villanovian culture spread across the peninsula. From the eighth century BC, Iron Age material culture developed a strong regional character. Distinct archaeological cultures evolved in areas that would later coincide with those occupied by historical populations: Etruscans, Venetians, Rhaetians, etc. [57, 89]. Population centres in the southern Po Plain, and later Veneto, grew larger and become more organised, with streets, rectilinear plans, and public buildings [58, 91]. Social stratification and the emergence an aristocracy visible is in the funerary record [57, 92]. Exchange relationships within the Po Plain intensified, as did trade with Europe, central Italy, and the Greek world, mediated by the region’s Etruscan cities [93].

Archaeobotanical remains from across northern Italy suggest a degree of continuity in crop choice between the Bronze and Iron Ages, with a potential increase in the importance of legumes [94]. The greater diffusions of iron, especially from the sixth century BC, expanded the use of metal ploughs and agricultural tools, and the potential to work heavier soils [95]. It has recently been proposed that surplus cereal production in the Iron Age Germany was achieved through manuring and an expansion in cultivation [96]. However, the applicability of this conclusion to an Italian lowland context is unclear. Pollen spectra do not indicate significant clearing during the Iron Age, e.g. [68], although the diffusion of iron tools and ploughs may have allowed cultivation of heavier soils that were already more or less free of trees. By the mid first millennium BC vine cultivation had become well established [97, 98], and in Etruscan territory it was sufficiently organised to produce a surplus for export [99–101]. This scale of production suggests established land-holding and long-term strategies in land use.

Urban centres continued to develop in the Veneto into the latter half of the millennium, but Etruscan influence decreased as a result of Roman expansion in the south and migrations from the north. Material evidence for La Tène culture in northern Italy expanded from the sixth century BC, and came to dominate large parts of the region by the fourth century BC [102, 103]. Although described by Roman authors as a violent invasion, the archaeological evidence presents a more complex picture of acculturation following the migration of ‘Celtic’ populations into northern Italy [104]. Etruscan cities that once flourished were abandoned in the third century BC, and the Roman Republic advanced into the region and gained control over the area between the third and second centuries BC [55, 105].

## Materials and methods

### Sites and assemblages

Late prehistoric sites from a large portion of the eastern Po Valley and Venetian Plain were chosen for this study (S1 Table). The study area was limited to these recent valleys and their borders, in order to compare sites subject to similar climatic conditions. In order to focus on the economic use of animals, only assemblages from domestic settlement contexts were considered in analysis of taxon presence/abundance: sanctuaries, cult sites, and ritual deposits were excluded. Ritual assemblages often have a different distribution of taxa from those found in domestic contexts, e.g. a pronounced focus on a single species [35] (e.g. [106, 107]), which could bias results. Sites are divided into regional groups based on their location within the study area and regional similarities in material culture. Faunal samples with very long chronologies that could not be assigned to a useful temporal range were excluded. Only dated contexts were analysed; surface finds and materials recovered from plowzone were not considered. Despite efforts for good regional and temporal coverage, faunal assemblages are not evenly distributed across the study areas and time periods. The assemblages also vary greatly in size, from a few dozen to tens-of-thousands of bones. Information on some periods is entirely lacking, while others are represented only by a single site. This large range results from differences in site type, degree of preservation, ancient settlement patterns, and historical interests. Modern development obscures Iron Age sites in particular, as many major settlements of this period are buried under modern–as well as medieval and Roman–cities. Although dividing the study area into smaller sub-regions limited the size of comparative samples, such division was necessary for investigating differences within the Po Basin. Illustrations and tables have been organised to clearly demonstrate where gaps are present.

### Quantifying taxonomic abundance and animal size

The relative proportions of cattle (*Bos taurus*), sheep/goat (*Ovis aries*/*Capra hircus*), and pigs (*Sus scrofa*) were quantified using the number of identified specimens (NISP). Although subject to several systematic biases, NISP offers a widely employed and easy to calculate method of assessing the relative frequency of different species [108]. Inter-observer differences in identification and recording are a primary concern [109–111], as they artificially inflate the importance of taxa with a greater number of ‘identifiable’ remains. In order to better control of differences in recording practice and identification skill, rib, and vertebrate fragments were excluded from NISP counts where possible, as were any specimens not identified as a specific skeletal element. Complete and partial skeletons were also excluded from these analyses, since their inclusion would inflate NISP counts. In order to apply statistical tests with a reasonable degree of confidence, only assemblages with a minimum of 110 specimens identified to the three main domesticates were considered in NISP analyses. The statistical significance of inter-regional differences was tested using a chi-squared test in R software [112].

Changes in animal size were assessed using size-index scaled Log Standard Index values (LSI) [113]. Widely available standards were used for cattle [114], sheep and goats [115], and pigs [116]. Log ratios offer a useful method for investigating animal size, because they allow measurements from different elements to be pooled into a larger sample of indices for each dimension (length and breadth). However, this method of grouping can lead to the overrepresentation of specimens that yield several measurements, a problem that is quickly compounded if a skeleton or articulating limb is included. To avoid over representation of articulating remains, our LSI analyses included only one measurement per specimen and one specimen from an articulating group of bones. Bone widths were considered separately from lengths in order to assess two-dimensional change. Measurements of bone depth were comparatively rare and were not included.

Table 1 presents the measurements used in LSI analyses in preferential order. Only one length or breadth measurement per specimen was considered, e.g. if GLl was available for a specimen, all subsequent length measurements would be excluded. This list purposefully excludes elements and measurements particularly sensitive to animal age, although it is also constrained by measurements included in the published standards. LSI values for cattle, sheep, and pigs are presented in histograms. Because accurate species distinction can be challenging for closely related taxa like sheep and goats [117], related species are also included: figures for sheep also display counts and means for goats and undistinguished sheep/goat specimens; figures for pigs also contain data identified as wild boar. Undistinguished *Sus* sp. were grouped with domestic pigs. The Mann-Whitney U Test, conducted in R, was used to identify statistically significant differences between populations. Only LSI values from cattle, sheep, and pig were subject to statistical analysis; goats were excluded due to a lack of reliable biometric information.

**Table 1:** Measurements used in calculation of LSI values for each taxon.

Dimension	Measurement	Reference	Cattle	Sheep/goat	Pig
Length	GL	von den Driesch 1976 [118]	Humerus, Radius, Mc, Mt, Calcaneum	Humerus, Radius, Mc, Mt, Femur, Tibia, Calcaneum	Mc III, Mc IV, Calcaneum
	GLl	von den Driesch 1976 [118]	Astragalus	Astragalus	Astragalus
	GLm	von den Driesch 1976 [118]	Astragalus		Astragalus
	HTC	Davis 1996 [115]	Humerus	Humerus	Humerus
	HT	Davis 1996 [115]		Humerus	
Breadth	Bd	von den Driesch 1976 [118]	Humerus, Radius, Mc, Mt, Tibia, Astragalus	Mc, Mt, Astragalus, Tibia	Humerus, Tibia
	BT	von den Driesch 1976 [118]	Humerus	Humerus	Humerus
	Bp	von den Driesch 1976 [118]	Humerus, Radius, Mc, Mt, Tibia	Radius, Mc	Radius
	BFd	von den Driesch 1976 [118]	Radius		
	BFp	von den Driesch 1976 [118]	Radius	Radius	

Mc = Metacarpal. Mt = Metatarsal.

Metacarpal measurements were used to investigate diachronic changes in sex ratios for cattle and sheep. The balance of males, females, and castrates within a population can influence observed variation in animal biometry, especially if the sexual composition of the group changed over time. Metacarpals are the most sexually dimorphic element in domestic bovids [119–121], and consideration of metacarpal size and shape can provide information on sex representation [122, 123]. To aid interpretation, measurements were compared to data from animals of known sex: archaeological cattle from Eketorp ringfort [123], identified using DNA, and modern Shetland sheep [115, 124].

## Results: Relative abundance of livestock

### Regional patterns

**Fig 2:**
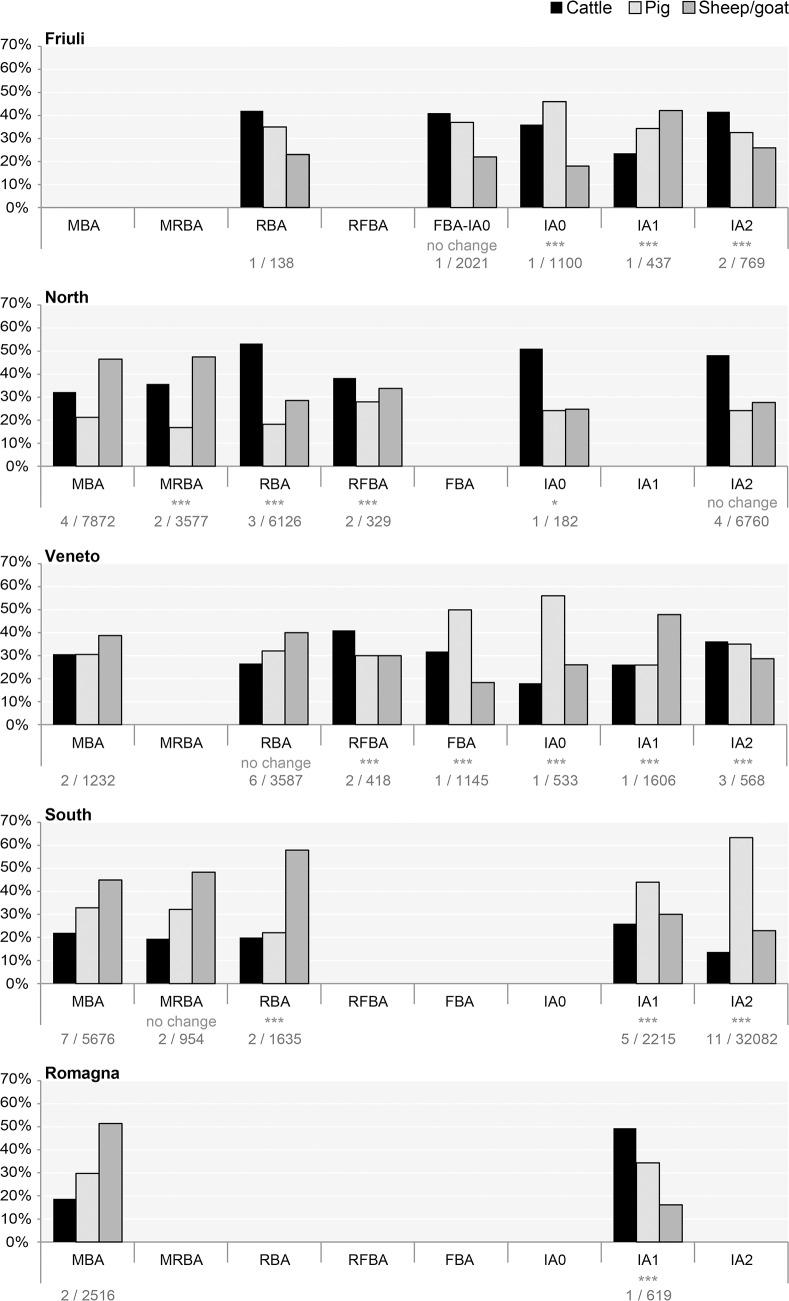
Relative percentages of principal domestic livestock. See Table 2 for sample size. Stars indicate significance of change from previous period according to Chi square tests: p ≤ 0.05*, p ≤ 0.01**, p ≤ 0.001*** (S2 Table).

Analysis of NISP data for taxon abundance and livestock ratios (Table 2) demonstrated that livestock representation varied significantly between regions (Fig 2). Inter-regional comparisons within each chronological period were statistically significant in nearly all instances, and these differences persisted through time (S2 Table). Amongst sites of the North group, pigs were relatively rare compared to cattle and sheep/goat. The proportions of livestock were more balanced between the three taxa in the Veneto, with the exception of FBA and IA0. Caprines and pigs were very abundant in the South study area, while cattle were less common. Livestock ratios from the Friuli and Romagna regions were more variable, which may reflect either a more heterogeneous approach to husbandry practices or variation resulting from the lower number of sites from these areas.

**Table 2:** NISP and relative percentages of principal livestock by study area.

Study area	Period		n. of assemblages	Mammal NISP	NISP 3 dom.	Cattle	Pig	Sheep /goat
Friuli	Middle Bronze Age	MBA						
	Middle-Recent Bronze Age	MRBA						
	Recent Bronze Age	RBA	1	144	138	42%	35%	23%
	Recent-Final Bronze Age	RFBA						
	Final Bronze Age-Early Iron Age	FBA-IA0	1	2106	2021	41%	37%	22%
	Early Iron Age	IA0	1	1153	1100	36%	46%	18%
	Mid Iron Age	IA1	1	451	437	24%	34%	42%
	Late Iron Age	IA2	2	813	769	42%	33%	26%
North	Middle Bronze Age	MBA	4	8714	7872	32%	21%	47%
	Middle-Recent Bronze Age	MRBA	2	3845	3577	36%	17%	47%
	Recent Bronze Age	RBA	3	6864	6126	53%	18%	29%
	Recent-Final Bronze Age	RFBA	2	352	329	38%	28%	34%
	Final Bronze Age	FBA						
	Early Iron Age	IA0	1	202	182	51%	24%	25%
	Mid Iron Age	IA1						
	Late Iron Age	IA2	4	7133	6760	48%	24%	28%
Veneto	Middle Bronze Age	MBA	2	1380	1232	31%	31%	39%
	Middle-Recent Bronze Age	MRBA						
	Recent Bronze Age	RBA	6	4015	3587	27%	32%	40%
	Recent-Final Bronze Age	RFBA	2	475	418	41%	30%	30%
	Final Bronze Age	FBA	1	1426	1145	32%	50%	18%
	Early Iron Age	IA0	1	781	533	18%	56%	26%
	Mid Iron Age	IA1	1	1714	1606	26%	26%	48%
	Late Iron Age	IA2	3	645	568	36%	35%	29%
South	Middle Bronze Age	MBA	7	6374	5676	22%	33%	45%
	Middle-Recent Bronze Age	MRBA	2	1051	954	19%	32%	48%
	Recent Bronze Age	RBA	2	1738	1635	20%	22%	58%
	Recent-Final Bronze Age	RFBA						
	Final Bronze Age	FBA						
	Early Iron Age	IA0						
	Mid Iron Age	IA1	5	2387	2215	26%	44%	30%
	Late Iron Age	IA2	11	34168	32082	14%	63%	23%
Romagna	Middle Bronze Age	MBA	2	2800	2516	19%	30%	51%
	Mid Iron Age	IA1	1	648	619	49%	34%	16%

### Diachronic change

Study regions both north and south of the River Po displayed major diachronic changes in livestock representation, but each area followed a distinct trajectory. Within individual regions, diachronic change was statistically significant for most temporal transitions (indicated in Fig 2; S2 Table). Patterns of animal exploitation evolved in all areas, but the direction and timing of these changes differed. South of the Po River, a shift in livestock percentages is apparent in the Iron Age. Its origin is obscured by a lack of data for FBA and IA0, but significant changes to livestock ratios occurred in both the South and Romagna regions by IA1. Pigs replaced sheep/goat as the most abundant taxon in the South group; caprines also decreased in the Romagna area, but there cattle rose in importance. North of the Po River, changes to NISP percentages occurred during RBA, earlier than in the southern plain. As in the other regions, the relative abundance of sheep/goat decreased, with cattle replacing them as the dominant taxon. This emphasis on cattle exploitation continued in subsequent periods, albeit to a lesser extent during RFBA–variation that could result from the small sample size. `

Unlike regions with a single change in species distribution, Friuli and the Veneto presented several fluctuations in NISP percentages. After an emphasis on sheep/goat husbandry in MBA and RBA, NISP data from the Veneto varied across the remainder of the Bronze Age and the Iron Age. For certain periods few sites were available. This factor probably contributes to the observed variation: during FBA and IA0, the Veneto is represented by a single site. This settlement, Frattesina, was a unique proto-urban centre with far-reaching trade networks [90]. Its NISP profile, with a large percentage of pigs, more closely resembles the Etruscan/Roman pattern than that of the Bronze Age sites considered here. Of all the regions, NISP patterns in Friuli are the most variable, potentially as a result of a low number of available assemblages.

## Results: Biometry

Analysis of livestock biometry utilised 4886 unique post-cranial measurements. Table 3 presents summary statistics of LSI values by region and period. As stated above, the statistical significance of temporal and inter-regional differences were analysed using the Mann-Whitney U tests (S3 Table). The significance of diachronic changes is indicated in the relevant histograms; full results are available in S3 Table.

**Table 3:** Sample size, mean, and standard deviation of LSI values per area and time period.

			Length						Width					
Taxon	Region	Period	n	n sites	min	max	mean	sd	n	n sites	min	max	mean	sd
Cattle	Romagna	BM-R	31	2	-0.0640	0.0350	-0.0180	0.0252	35	3	-0.0785	0.0916	0.0064	0.0400
Cattle	Romagna	IA1	9	1	-0.0481	0.0400	-0.0024	0.0281	37	1	-0.1605	0.1264	0.0309	0.0551
Cattle	Friuli	BM-R							3	1	-0.1239	-0.0642	-0.0997	0.0314
Cattle	Friuli	BRF-IA0	51	3	-0.0764	0.0463	0.0008	0.0287	104	4	-0.1239	0.1335	0.0502	0.0502
Cattle	Friuli	IA1	1	1	0.0052	0.0052	0.0052	NA	4	1	0.0171	0.0693	0.0425	0.0227
Cattle	Friuli	IA2	1	1	-0.0351	-0.0351	-0.0351	NA	7	2	0.0088	0.0990	0.0466	0.0382
Cattle	North	BM-R	159	8	-0.1287	0.0600	-0.0176	0.0295	295	9	-0.1777	0.1264	0.0056	0.0533
Cattle	North	BRF-IA0	3	2	-0.0350	0.0252	-0.0061	0.0302	8	2	-0.0683	0.0760	0.0194	0.0502
Cattle	North	IA2	79	3	-0.0695	0.0794	-0.0043	0.0326	161	3	-0.0798	0.1601	0.0250	0.0494
Cattle	South	BM-R	49	8	-0.0778	0.0388	-0.0205	0.0278	107	8	-0.1096	0.1196	-0.0079	0.0526
Cattle	South	IA1	7	3	-0.0560	0.0144	-0.0195	0.0244	15	5	-0.0168	0.1027	0.0524	0.0364
Cattle	South	IA2	78	11	-0.0706	0.0869	0.0084	0.0316	134	14	-0.0558	0.1733	0.0508	0.0497
Cattle	Veneto	BM-R	34	7	-0.1032	0.0283	-0.0303	0.0341	92	7	-0.1581	0.1175	-0.0247	0.0489
Cattle	Veneto	BRF-IA0	6	1	-0.0600	0.0124	-0.0300	0.0288	15	1	-0.0896	0.0901	0.0015	0.0423
Cattle	Veneto	IA1	16	2	-0.0756	0.0640	-0.0034	0.0397	33	2	-0.1160	0.1453	0.0175	0.0624
Cattle	Veneto	IA2	2	2	-0.0282	0.0312	0.0015	0.0420	3	2	-0.0220	0.0751	0.0181	0.0507
Sheep	Romagna	BM-R	30	2	-0.0705	0.0759	0.0026	0.0331	51	2	-0.1135	0.0589	-0.0133	0.0320
Sheep	Romagna	IA1	2	1	0.0286	0.0408	0.0347	0.0086	5	1	-0.0445	0.0470	0.0152	0.0367
Sheep	Friuli	BRF-IA0	22	3	-0.0546	0.1317	0.0543	0.0450	38	3	-0.0188	0.1256	0.0374	0.0351
Sheep	Friuli	IA1	3	1	0.0745	0.0761	0.0755	0.0008	4	1	0.0454	0.0785	0.0655	0.0144
Sheep	Friuli	IA2	2	2	0.0477	0.0761	0.0619	0.0201	2	1	0.0069	0.0768	0.0418	0.0494
Sheep	North	BM-R	131	7	-0.1847	0.0762	0.0038	0.0440	158	6	-0.1657	0.0934	-0.0270	0.0378
Sheep	North	BRF-IA0	3	2	0.0032	0.0559	0.0298	0.0263	6	2	-0.0417	0.0215	-0.0042	0.0233
Sheep	North	IA2	52	3	-0.0067	0.1078	0.0370	0.0257	102	3	-0.0410	0.1000	0.0296	0.0284
Sheep	South	BM-R	26	5	-0.0724	0.0588	-0.0068	0.0366	49	7	-0.0969	0.1268	-0.0279	0.0354
Sheep	South	IA2	103	3	-0.0369	0.1129	0.0341	0.0297	158	5	-0.0516	0.0931	0.0158	0.0275
Sheep	Veneto	BM-R	20	5	-0.0566	0.0710	0.0274	0.0339	53	6	-0.1154	0.0802	0.0049	0.0462
Sheep	Veneto	BRF-IA0	4	1	0.0206	0.0604	0.0322	0.0188	10	1	-0.0231	0.0721	0.0185	0.0306
Sheep	Veneto	IA1	30	1	0.0175	0.1265	0.0660	0.0270	70	1	-0.0506	0.1021	0.0403	0.0278
Goat	Romagna	BM-R	2	2	-0.0648	0.0495	-0.0076	0.0808	4	2	-0.0754	0.1430	0.0063	0.0974
Goat	Romagna	IA1							2	1	-0.0430	0.0220	-0.0105	0.0460
Goat	Friuli	BRF-IA0	1	1	0.0138	0.0138	0.0138	NA	4	1	0.0680	0.1255	0.0960	0.0323
Goat	North	BM-R	28	5	-0.1490	0.1699	0.0182	0.0609	41	6	-0.1154	0.1177	0.0093	0.0457
Goat	North	IA2	11	3	-0.0016	0.0620	0.0368	0.0185	14	3	0.0098	0.0836	0.0343	0.0237
Goat	South	BM-R	6	2	-0.0200	0.0350	0.0145	0.0201	6	2	-0.0809	0.0296	-0.0172	0.0387
Goat	South	IA2	21	4	-0.0234	0.0924	0.0315	0.0342	28	4	-0.0075	0.1322	0.0405	0.0307
Goat	Veneto	BM-R	2	1	-0.0070	0.0049	-0.0011	0.0084	6	3	-0.0479	0.0429	0.0029	0.0424
Goat	Veneto	IA2							1	1	0.0466	0.0466	0.0466	NA
Sheep/goat	Romagna	BM-R	1	1	-0.0083	-0.0083	-0.0083	NA	7	1	-0.0798	0.0206	-0.0164	0.0364
Sheep/goat	Romagna	IA1							11	1	-0.0672	0.0252	-0.0296	0.0266
Sheep/goat	Friuli	BRF-IA0	6	1	0.0206	0.0506	0.0346	0.0136	6	2	0.0019	0.0969	0.0417	0.0372
Sheep/goat	Friuli	IA2							1	1	-0.0017	-0.0017	-0.0017	NA
Sheep/goat	North	BM-R	18	4	-0.1810	0.0256	-0.0519	0.0656	62	5	-0.0986	0.0539	-0.0192	0.0318
Sheep/goat	North	BRF-IA0							2	2	-0.0035	0.0361	0.0163	0.0280
Sheep/goat	North	IA2	1	1	0.0289	0.0289	0.0289	NA	2	2	0.0109	0.0506	0.0307	0.0281
Sheep/goat	South	BM-R	10	4	-0.0749	0.0506	-0.0121	0.0346	56	6	-0.0880	0.0743	-0.0171	0.0327
Sheep/goat	South	IA1	4	1	-0.0082	0.0771	0.0254	0.0368	13	3	-0.0553	0.0536	0.0096	0.0256
Sheep/goat	South	IA2	61	7	-0.0417	0.1380	0.0256	0.0322	69	10	-0.0711	0.1361	0.0118	0.0342
Sheep/goat	Veneto	BM-R	8	1	-0.1064	-0.0034	-0.0555	0.0308	29	5	-0.1316	0.1322	-0.0142	0.0581
Sheep/goat	Veneto	BRF-IA0	1	1	0.0854	0.0854	0.0854	NA	1	1	0.0726	0.0726	0.0726	NA
Sheep/goat	Veneto	IA1	1	1	0.0718	0.0718	0.0718	NA	17	1	-0.0693	0.1122	0.0253	0.0499
Sheep/goat	Veneto	IA2	1	1	0.0222	0.0222	0.0222	NA	1	1	0.0073	0.0073	0.0073	NA
Pig	Romagna	BM-R	8	1	-0.0390	0.0257	-0.0170	0.0207	14	2	-0.1311	0.0474	-0.0391	0.0451
Pig	Romagna	IA1	12	1	-0.0615	-0.0044	-0.0283	0.0194	20	1	-0.0888	0.0094	-0.0281	0.0248
Pig	Friuli	BRF-IA0	7	2	-0.0544	0.0400	-0.0062	0.0290	5	3	-0.0624	0.0371	-0.0114	0.0416
Pig	Friuli	IA1	1	1	0.0021	0.0021	0.0021	NA	13	1	-0.1367	0.0251	-0.0409	0.0389
Pig	Friuli	IA2	4	2	-0.0484	-0.0332	-0.0425	0.0067	5	2	-0.0447	0.0326	-0.0029	0.0355
Pig	North	BM-R	36	8	-0.0678	0.1417	0.0084	0.0501	58	6	-0.1124	0.0894	-0.0348	0.0361
Pig	North	BRF-IA0	2	1	-0.0544	-0.0229	-0.0387	0.0222	1	1	-0.0600	-0.0600	-0.0600	NA
Pig	North	IA2	41	3	-0.0741	0.1252	-0.0294	0.0350	43	3	-0.1020	0.0808	-0.0353	0.0377
Pig	South	BM-R	22	7	-0.0654	0.0348	-0.0031	0.0266	58	6	-0.0892	0.1186	-0.0255	0.0355
Pig	South	IA1	7	3	-0.1666	-0.0174	-0.0655	0.0474	21	4	-0.0914	0.0588	-0.0391	0.0351
Pig	South	IA2	676	8	-0.1082	0.1409	-0.0280	0.0352	415	11	-0.1745	0.1022	-0.0408	0.0364
Pig	Veneto	BM-R	19	5	-0.1184	0.0328	-0.0247	0.0363	41	7	-0.1225	0.0326	-0.0446	0.0406
Pig	Veneto	BRF-IA0	1	1	-0.0662	-0.0662	-0.0662	NA	5	1	-0.0722	-0.0032	-0.0383	0.0248
Pig	Veneto	IA1	3	1	-0.0173	0.0624	0.0305	0.0422	12	1	-0.0773	0.0713	-0.0020	0.0568
Pig	Veneto	IA2	2	1	-0.0459	-0.0445	-0.0452	0.0010						
Wild boar	Romagna	BM-R	3	2	0.0964	0.1208	0.1089	0.0122						
Wild Boar	Romagna	IA1							1	1	0.0869	0.0869	0.0869	NA
Wild boar	Friuli	BRF-IA0	1	1	0.1165	0.1165	0.1165	NA						
Wild Boar	Friuli	IA1	1	1	0.1029	0.1029	0.1029	NA	1	1	0.1382	0.1382	0.1382	NA
Wild Boar	North	BM-R	13	2	0.0550	0.1492	0.1097	0.0282	8	3	-0.0331	0.1788	0.0923	0.0809
Wild boar	North	BRF-IA0	1	1	0.1257	0.1257	0.1257	NA						
Wild Boar	North	IA2	2	1	0.0942	0.1252	0.1097	0.0220	4	1	0.0692	0.1084	0.0925	0.0178
Wild Boar	South	BM-R	5	2	0.0416	0.1269	0.0931	0.0320	5	2	0.0834	0.2137	0.1451	0.0483
Wild Boar	South	IA2	2	2	0.0503	0.0900	0.0702	0.0281	3	2	0.0834	0.2017	0.1323	0.0617
Wild Boar	Veneto	BM-R	1	1	0.1093	0.1093	0.1093	NA	4	3	-0.1225	0.0892	-0.0282	0.0876
Wild boar	Veneto	IA1	2	1	0.1144	0.1318	0.1231	0.0123						

### Cattle

#### Diachronic change

**Fig 3:**
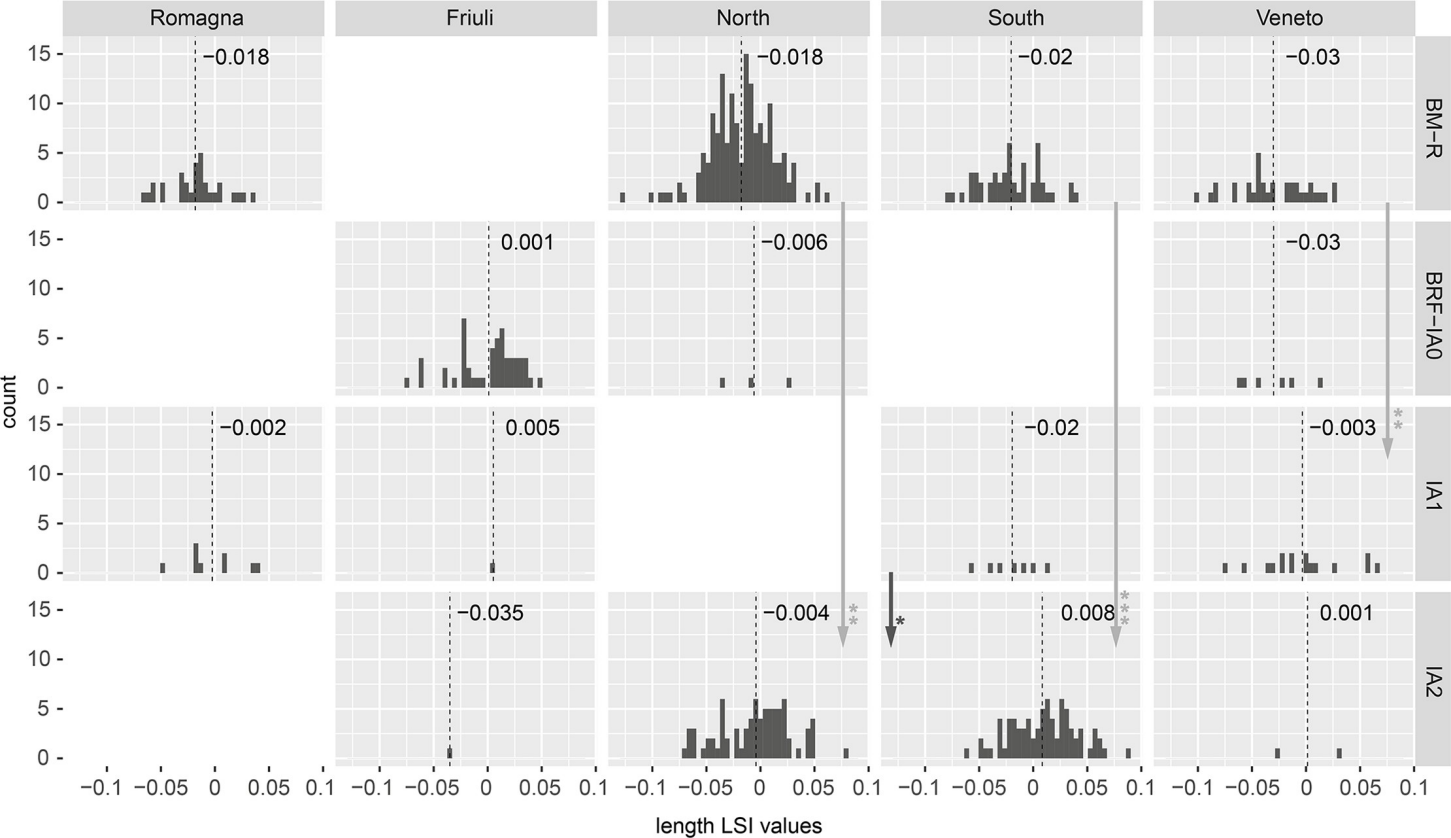
Distribution of cattle bone length LSI values by study region and period. Arrows indicate significant diachronic changes according to Mann-Whitney U tests (S3 Table).

LSI values from cattle bone lengths (Fig 3) revealed statistically significant diachronic changes in the majority of study regions. Except in Friuli, for which there was too little data to draw conclusions, mean cattle lengths increased in all regions between the Bronze and Iron Ages. For the North and South study areas, the increase in bone lengths was most significant when BM-R and IA2 were compared; however, small increases in mean LSI values during BRF-IA0 and IA1 suggest that these changes were incremental. In the Veneto study region a significant increase in bone length occurred between BM-R and IA1. The Romagna region also displays a diachronic increase in average LSI lengths, although this change was not statistically significant (see S3 Table).

**Fig 4:**
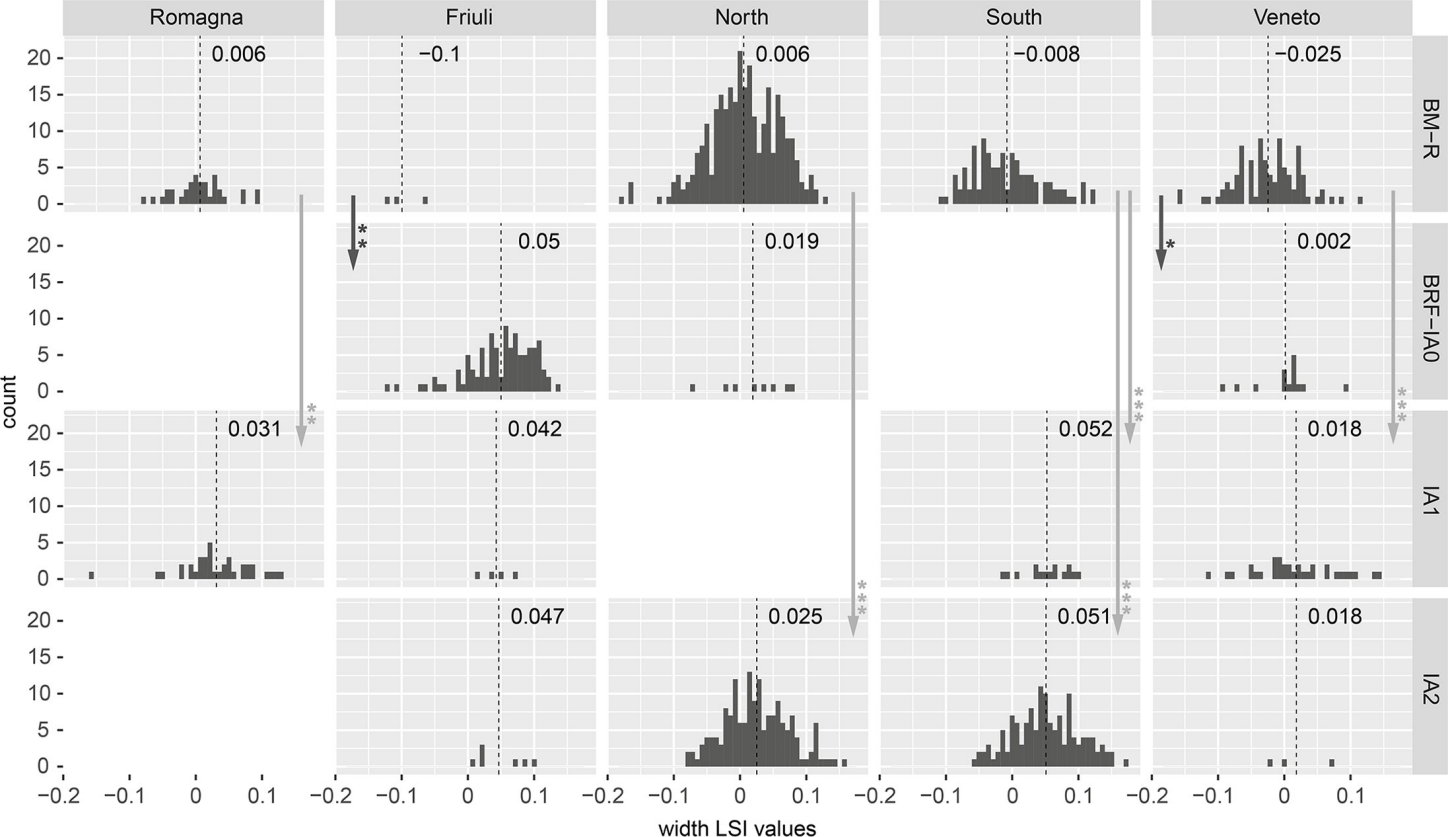
Distribution of cattle bone width LSI values by study region and period. Arrows indicate significant diachronic changes according to Mann-Whitney U tests (S3 Table).

Cattle LSI width values (Fig 4) also increased in size, although the timing of these changes did not consistently follow that of bone lengths. Width values from Friuli were extremely small in BM-R, but only three measurements were available. The Mann-Whitney U test demonstrated a significant size increase in BRF-IA0, but conclusions should be treated tentatively on account of the small BM-R sample size. Bone widths from the Romagna region increased between the BM-R and IA1; this increase was significant for widths but not for lengths. In the North study area, width LSI values behaved in a similar fashion to lengths: there was an incremental increase in the mean between BM-R and BRF-IA0, although this increase was only statistically significant when BM-R and IA2 were compared. Unlike other regions where lengths and width values grew in tandem, the two dimensions changed independently in the Veneto and South study areas. In the Veneto, a statistically significant increase in cattle widths is visible in the BRF-IA0, earlier than the change in cattle length values during IA1. Southern widths demonstrate a significant in increase in IA1, while cattle lengths in the region were relatively stable until IA2. In these two regions, an increase in bone width without comparable alteration in length would have produced a change in the shape of cattle, creating animals which were more robust, followed in the subsequent period by the emergence of taller and relatively more slender cattle.

#### Intra-period differences

LSI results demonstrated significant regional variation in the size of cattle, even within a single period. The data suggest that cattle in the Veneto were some of the smallest in northern Italy (in terms of animal dimensions), although data from Friuli were lacking: lengths from the Veneto were smaller on average than those from other areas during BM-R, although this difference was only statistically significant when compared to the North study area (p = 0.042*, see S3 Table). The mean of LSI widths from the Veneto was also lower than that of other BM-R regions, except for the 3 specimens from BM-R Friuli. Again, these differences were not statistically significant in all cases. Interestingly, after some sparse evidence for small animals during BM-R, Friuli produced data for large cattle during BRF-IA0. Lengths from the region during BRF-IA0 were significantly larger than any BM-R study area (p = 0.003**–0.000***) as well as other areas during BRF-IA0. Cattle LSI widths from Friuli are also significantly larger than those from other regions during the BM-R (p = 0.000***) and BRF-IA0 Veneto (p = 0.000***).

During IA2, the South study area contained the largest cattle, both in terms of individual measurements as well as for mean size. The Mann-Whitney U test demonstrated significant differences between Southern bone lengths (p = 0.010*) and widths (p = 0.000***) and those from Northern cattle in IA2. They also had greater LSI width values than Venetian cattle, at least during IA1. Interestingly, the samples from IA2 Southern cattle were not statistically different from BRF-IA0 cattle from Friuli, the largest animals of the Bronze Age.

#### Sex ratios

**Fig 5:**
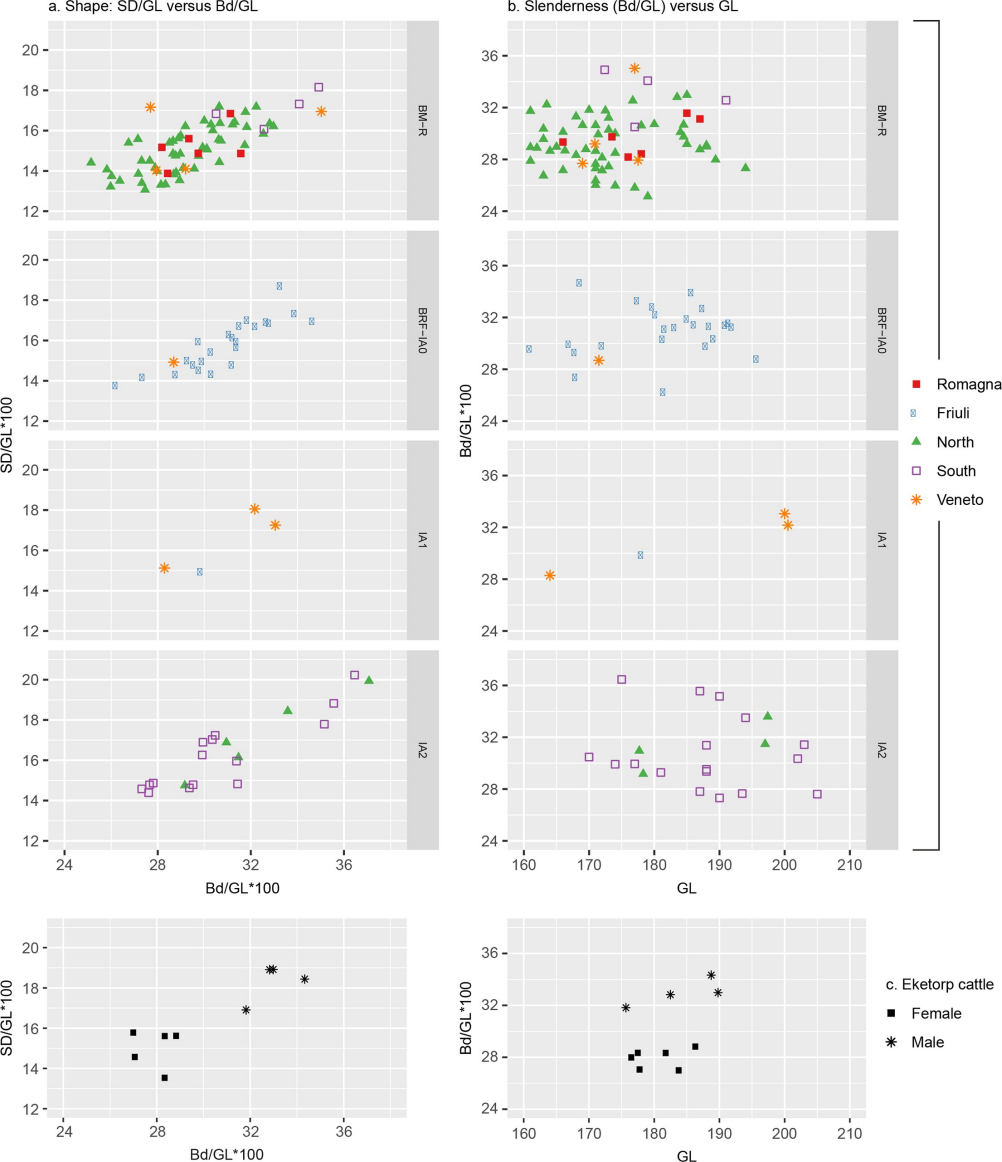
**Cattle metacarpal (a) shape, using slenderness indices (shaft width/length versus distal width/length), and (b) slenderness (shaft width/length) versus length.** Comparative data from Telldahl et al. [123].

Evaluation of the sex ratios within the study regions was difficult due to limited data. Consideration of cattle metacarpal shape (Fig 5A) did not produce a clear pattern of sexual dimorphism during the Bronze Age (e.g. North, Friuli); however, the large range of values–comparable with Eketorp ringfort [123]–suggests a mixed population comprised of males, females, and castrates. Subsequently, the appearance of very robust cattle in IA2 was accompanied by disappearance of the most slender individuals, alongside a clearer separation of slender and robust shape groups, indicating that both male and female cattle become more robust. Fig 5B suggests that some of the very tall IA2 cattle, particularly in the South, may have been castrates, on account of their long length and slender shape. Because the points lie on the established regression line, an internal change in sex ratios is more plausible than the introduction of another breed or type [122]. Overall, while changes to sex ratios may have impacted the observed size change, metacarpal data indicate an increase in height and robustness in both sexes, and thus general change in population size, potentially influenced by–but not only the result of–changes in sex composition.

### Sheep

#### Diachronic change

**Fig 6:**
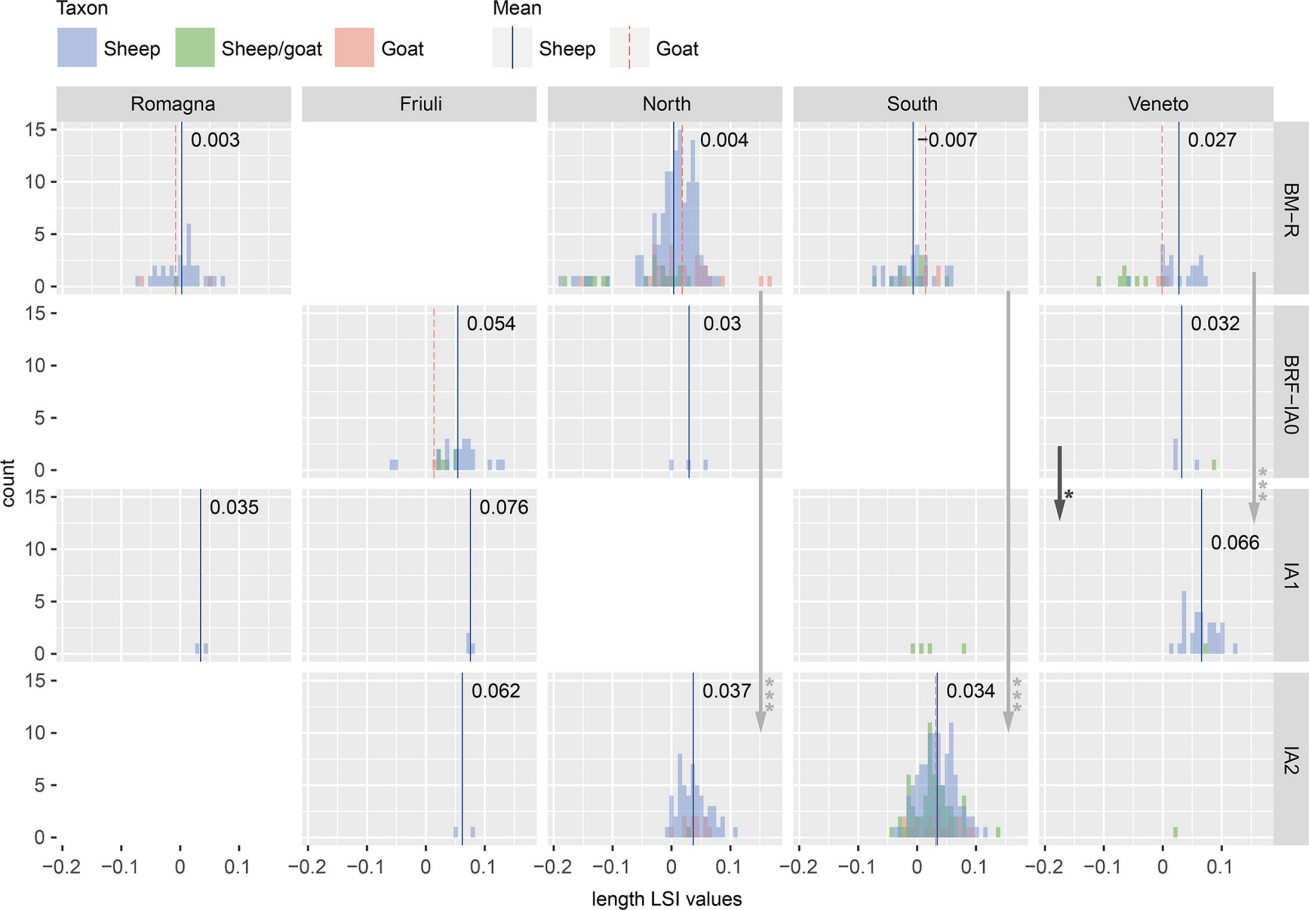
Distribution of sheep bone length LSI values by study region and period. Includes specimens identified as sheep, goat, and sheep/goat. Arrows indicate significant diachronic changes according to Mann-Whitney U tests (S3 Table) for sheep.

**Fig 7:**
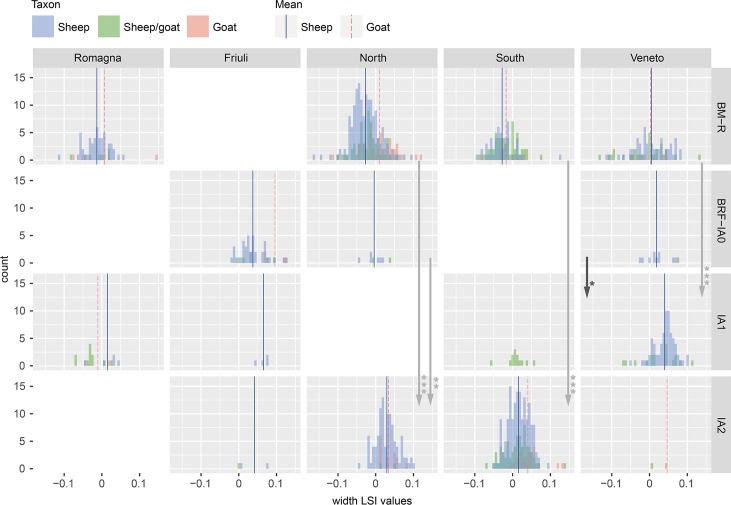
Distribution of sheep bone width LSI values by study region and period. Includes specimens identified as sheep, goat, and sheep/goat. Arrows indicate significant diachronic changes according to Mann-Whitney U tests (S3 Table) for sheep.

There were fewer measurements available for sheep than cattle, a result of the difficulty of distinguishing sheep from goats based on the morphology of their post-cranial remains [117]. Unlike cattle, changes in sheep bone lengths were typically mirrored by similar changes in widths. Between the Bronze and Iron Ages, mean LSI values from sheep increased in size in all regions (Figs 6 and 7). In Friuli and Romagna study areas, this change was not statistically significant (see S3 Table). In the North, the change in mean was incremental, with statistical differences present in a comparison of BM-R and IA2, as well as BRF-IA0 to IA2 widths. In the South, sheep measurements were only available for the first and final time periods, between which a significant change in both dimensions was apparent. An incremental increase in mean was present in LSI values for both dimension from the Veneto, but statistical differences were only noted for the change to IA1.

#### Intra-period differences

Sheep of the same temporal period differed in size across the study regions. Like for cattle, mean LSI values for sheep from Friuli were larger than contemporary animals in other study areas. Compared to animals from the BM-R, LSI values from BRF-IA0 Friuli were significantly larger in all length (p = 0.015*–0.000***) and width (p = 0.001***–0.000***) comparisons. BM-R sheep from the Veneto were also comparatively large, with significant differences in length and width values compared to those in the Romagna, North, and South study areas. These regional size differences appear to have persisted into the Iron Age, although the lack of data from Friuli complicates this assessment. IA2 sheep in the North and South were of a similar height on average, but LSI width values from the South were significantly smaller (p = 0.0007***). Southern animals were therefore of a comparable height as those in the North, but more slender.

#### Sex ratios

**Fig 8:**
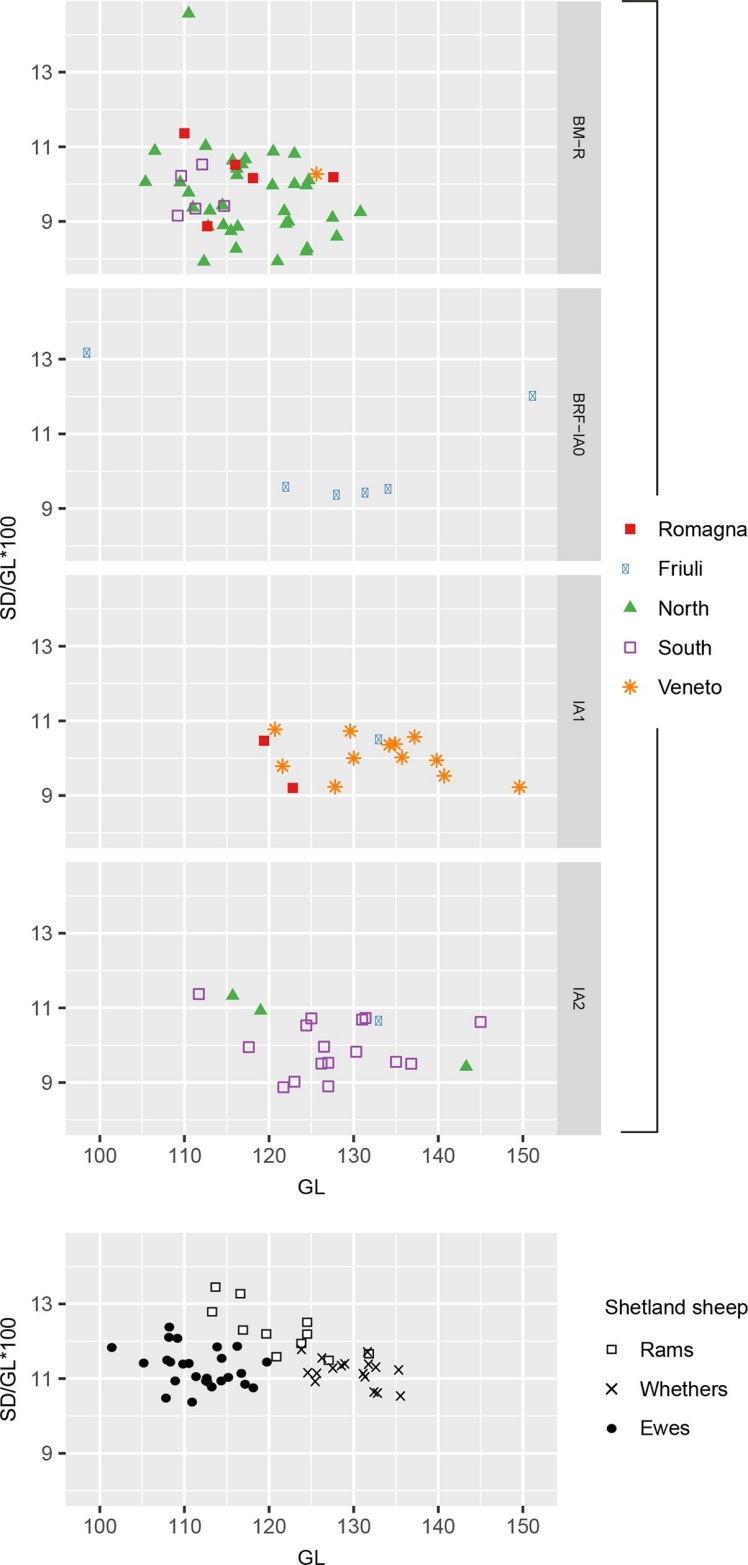
Sheep metacarpal slenderness (shaft width/length) versus length. Comparative data from Davis [115, 124].

Data from sheep metacarpals (Fig 8) indicates a shift to taller, more slender sheep. During the Iron Age, new tall animals emerge and the shortest and most slender drop out, suggesting development in the size/shape of the whole population and not only a change in sex ratios. This increase in tall and relatively slender animals could also reflect a shift in sex ratios to include more castrates; however, such a change would not be consistent with culling profiles from Iron Age sites south of the Po, which demonstrate an increase in lamb mortality after the Bronze Age (presumably focused on males). In some cases mortality in the first year reaches over 50% [35].

### Pigs

#### Diachronic change

**Fig 9:**
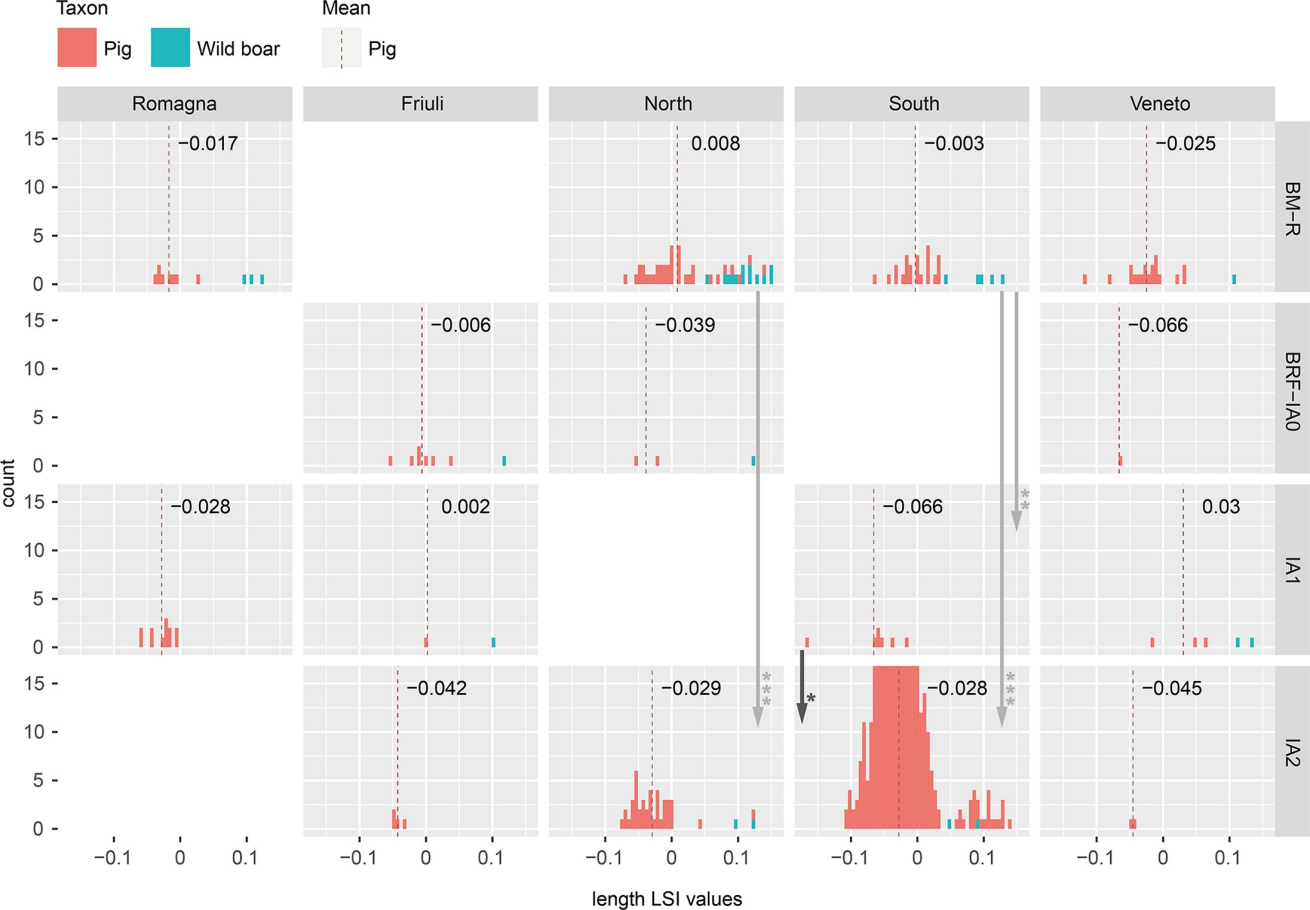
Distribution of pig bone length LSI values by study region and period. Includes specimens identified as domestic and wild pigs. Arrows indicate significant diachronic changes according to Mann-Whitney U tests (S3 Table) for domestic pigs.

**Fig 10:**
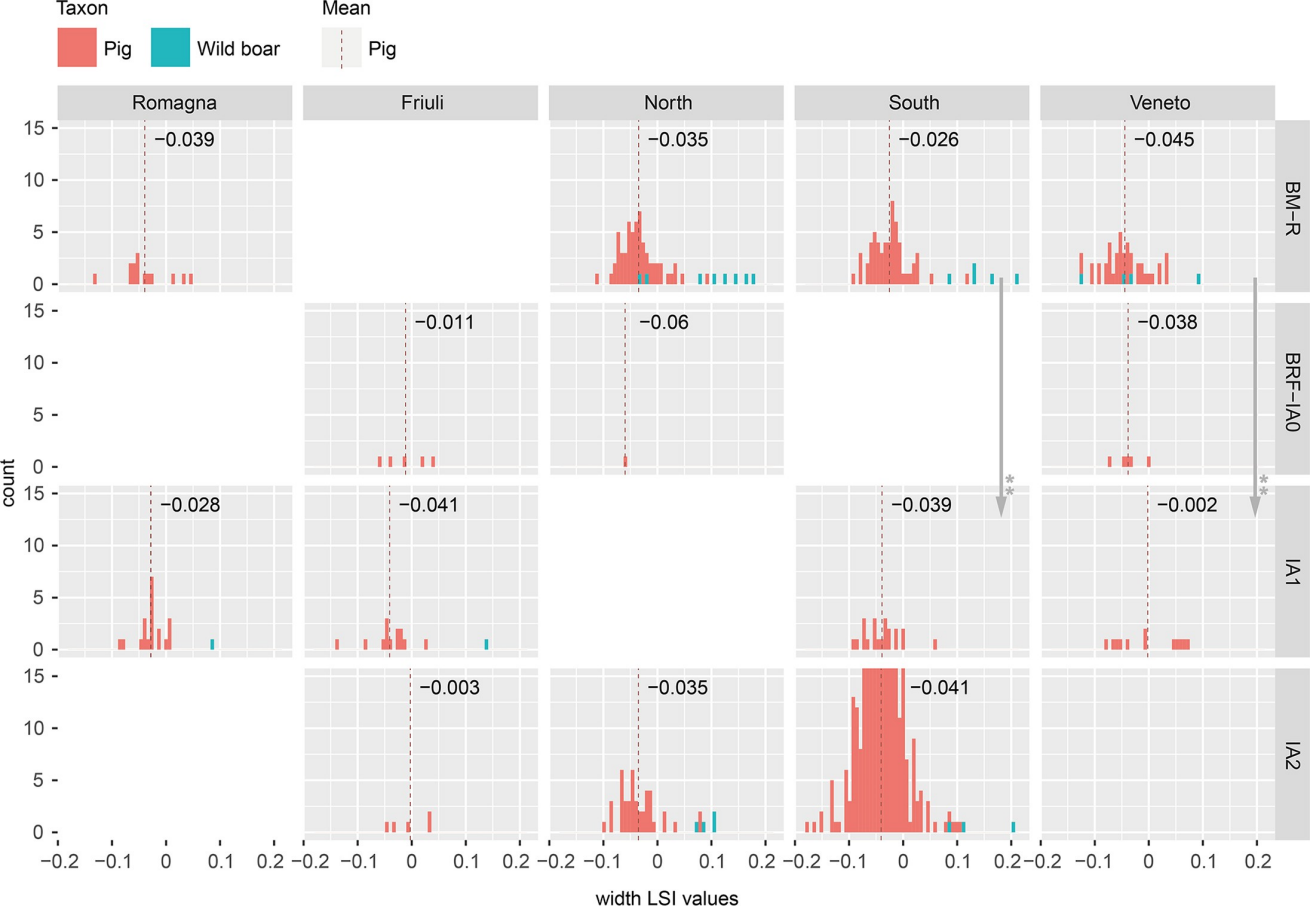
Distribution of pig bone width LSI values by study region and period. Includes specimens identified as domestic and wild pigs. Includes specimens identified as domestic and wild pigs. Arrows indicate significant diachronic changes according to Mann-Whitney U tests (S3 Table) for domestic pigs.

The complex relationship between domestic pigs and wild boar complicated analysis of pig biometry. Histograms of LSI values from pig length (Fig 9) and width (Fig 10) measurements demonstrate different levels of separation between populations of domestic pigs and larger wild boar. In some areas/periods, there was a distinct separation between the two populations, and specimen identifications conformed to the expected size of each species. In other study areas, LSI values from the two species were not so easily distinguished. This observed overlap between the species resulted from several factors: recording practices that did not distinguish between wild and domestic suids (grouped here with domestic pigs), misidentifications, and potentially inter-breeding between wild and domestic animals. For instance, the bi-model distribution of IA2 South LSI widths suggested a large population of domestic pigs alongside a small population of wild boar. Conversely, for BM-R North there was not a clear separation between the wild and domestic populations (regardless of zooarchaeological identification), possibly indicating of a greater level of genetic mixing between wild and domestic *Sus*.

In contrast to cattle and sheep, the predominant trend in domestic pigs LSI values was a reduction in bone lengths. Mean values for pig LSI lengths decreased in all regions between the Bronze and Iron Ages. This change was only statistically significant in the North and South study areas (see S3 Table), but the small samples available for regions could have precluded statistical identification of similar trends. Changes in pig widths were more variable. In the Romagna study area, the mean of LSI width values increased through time while average length decreased, suggesting that the pigs became shorter and more robust. In the North, width values remained constant while bone lengths decreased; here too pigs became shorter and relatively more robust. Few length measurements were available for the Veneto, precluding identification of changes in animal height, but pig bone widths significantly increased in size between BM-R and IA1. However, misidentification of wild boar may have influenced this analysis: the largest domestic LSI values for the Veneto are greater than 0.5, an approximate dividing line between domestic pigs and wild boar in other areas. In contrast to other regions, pigs in the South became smaller overall, and both width and length LSI values decreased significantly.

#### Intra-period differences

Like the other domestic taxa, pigs varied in size between regions. For BM-R, pig LSI length values were greatest in the North, while mean widths values were larger in the South. These differences were only statistically significant for a comparison of the South (lengths and widths, p = 0.040*, 0.016*) or North (lengths, p = 0.026*) areas with the Veneto. By IA2, the North and South study areas came to share similar mean length values. LSI lengths from IA1 in the Romagna study area were comparable to bone lengths from IA2 North and South. Widths from Romagna IA1 pigs were significantly larger than width values from the South study area (p = 0.023*), implying that pigs in Romagna were of a similar height to Southern animals, but more robust during the Iron Age. Too few data were available to draw conclusions about pig bone lengths from Veneto and Friuli. For pig LSI widths in Friuli, input from misidentified wild boar seems to have been a contributing factor in the larger size of animals.

### Summary of biometric data

With exception of samples containing very few measurements, mean LSI values from cattle and sheep increased in all regions between the Bronze Age and Iron Age. This increase was present in both bone widths and lengths, demonstrating an overall increase in animal size. These changes were statistically demonstrable for study areas with larger data sets: North, South, and Veneto. Consideration of metacarpal size/shape indicated that both males and females increased in size; biometric change was not the result of differences in sex ratios. Identification of the timing of this increase, and particularly of the earliest evidence for change, proved difficult due to the distribution of the data and the comparatively small quantity of measurements available for the important transitional period spanning the Final Bronze Age (BF) and first centuries of the Iron Age (IA0). Nevertheless, statistically significant changes to length/width LSI values of cattle and sheep were present for comparisons of previous period to IA1 in several study areas. Earlier and statistically significant increases in cattle width values were also visible in the Veneto in BRF-IA0. Also interesting is that mean LSI values for cattle and sheep increased between BM-R and BR-IA0 for all periods with data, except for Veneto cattle lengths. In only one instance was this increase statistically significant (Veneto cattle widths), but the small samples might have precluded statistical identification of real incremental change. LSI analysis also revealed differences in the nature of size change for different species. While sheep lengths and widths increased in tandem, cattle widths increased prior to cattle lengths in the South (IA1) and Veneto (BRF-IA0). Pigs displayed changes distinct from those of bovids. In study areas with larger samples (North, South, and Romagna), mean pig LSI lengths decreased, indicating that swine became shorter in these regions. In the South, a corresponding decrease in LSI width values occurred in the IA1. In other regions, width values were more variable: the mean increased in IA1 Veneto and Romagna study areas, although the inclusion of misidentified wild boar specimens or hybrids could have contributed to this trend.

## Discussion

### Patterns of species exploitation

**Fig 11:**
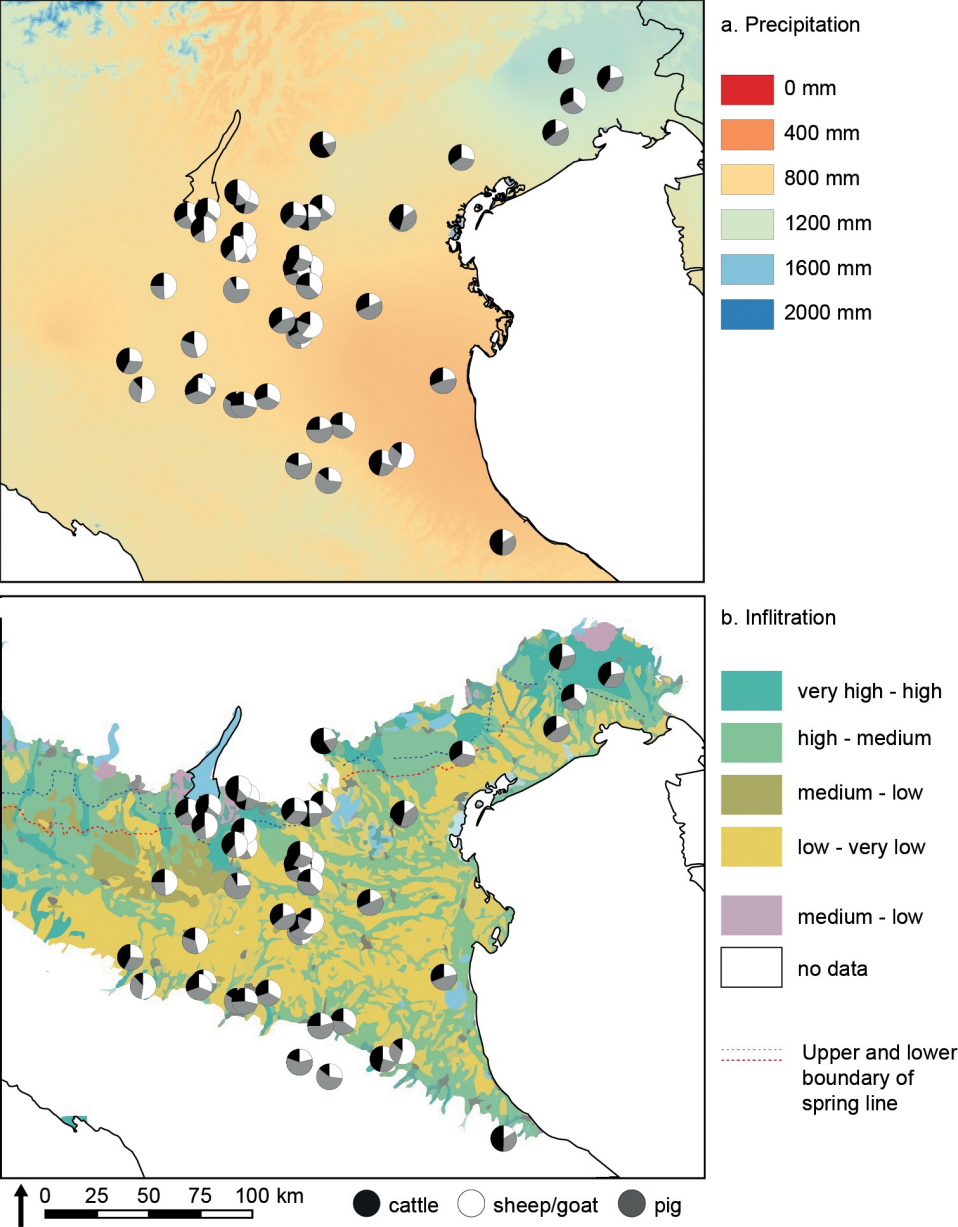
Maps of present-day precipitation and ground infiltration with relative percentages of the three main domesticates by site. Precipitation data from WorldClim 1.4 (current conditions) by www.worldclim.org [127]. Infiltration data follows Giuliano et al. [128].

Previous studies [7, 35, 46–50] have have provided syntheses of regional zooarcheaological data, but ours is the first to unify considertion of species frequencies and detailed biometric analysis over later prehistory across such a large part of northern Italy. Our results demonstrate a high degree of regionality in livestock ratios, which varied significantly between, and sometimes within, study areas, even during within a single chronological period. Consideration of site location (see Fig 1), precipitation and ground infiltration (Fig 11) demonstrates that rainfall and elevation did not have a universal impact on species frequencies. Friuli, a region with a cooler and wetter climate than the central Po Valley, contains a relatively high proportion of cattle, but the trend is not consistent across all periods, and cattle-dominant strategies were not constrained to the region. Data from individual sites also suggest diverse responses to high humidity: Fondo Paviani, which has produced evidence for a local shift to wetter conditions during the Final Bronze Age [125], saw a decrease in the abundance of cattle over the same period [126]. Elevation also did not predetermine patterns of exploitation: pre-Alpine sites preferred cattle, but pigs predominated on settlements in the Apennine range.

Geomorphology may have had a more significant role in shaping differences in animal management north and south of the River Po. The northern Po Valley contains greater areas of free-draining gravelly sediments, while the lower plain is characterised by silts and clays. South of Lake Garda, Pleistocene fluvial and fluvio-glacial alluvial deposits extend southward much of the way to the River Po, and similar deposits cover the northern Veneto and large areas of Friuli [129, 130]. The surface of the southern Po Plain is younger, and covered with fine Holocene fluvial deposits that stretch to pede-Apennine alluvial fans. As a result of these finer sediments and the shallow depth of the water table (currently c. 1.5–3 m from the surface), the lower plain is wetter and less permeable, promoting a damper landscape [131]. The different hydrology of these areas has modern agricultural implications, which promote or constrain various types and scales of irrigation and crop cultivation. Areas less suited to certain forms of plant agriculture may have adopted pastoral economic strategies. Equally, communities in free-draining areas may also have benefitted from access to a greater range of mobility routes, without the difficulty of navigating large areas of wet, low-lying territory, where flooding or marshy ground could impede the movement of large herds.

While local environmental conditions and resource availability might promote or preclude certain forms of production, the diverse diachronic changes in species frequencies demonstrate that cultural preferences and economic strategies also influenced livestock production (see also [45]). The significant expansion of pig production on Etruscan sites in the southern Po Valley is the clearest example of a culturally specific pattern of animal exploitation [cf. [51]). The increase in pork consumption in Etruscan territory has been related to socio-economic changes that encouraged surplus meat production, probably used to provision traders or re-distributed in service of political authority as tax or tribute [35]. By the sixth century BC, the abundance of pig bones on these sites probably represents a real intensification of swine husbandry–achieved, for example, through multiple farrowing induced by supplemental feeding–, as well as the movement of animals to central places. Such a strategy has been proposed for the Celtic village of Levroux in France (2nd century BC) [132]. Interestingly, Frattesina in the Veneto also contained a large abundance of pig remains. The identification of an ‘Etruscan’ NISP pattern on this site suggests that the origins of the preference for swine husbandry extend back to the early Iron Age or Final Bronze Age, and the beginning of the proto-Villanovan culture in northern Italy. Although located in the Veneto, the material culture of Frattesina also places the site within this cultural phenomenon of southern Po Plain, and links it directly with later central places like Etruscan Bologna, which succeeded Frattesina as a major centre of production and exchange [57, 133]. As a central place of major inter-regional importance, similar–if less intense pronounced–socio-economic incentives for surplus meat production would have existed at Frattesina, which has produced ample evidence for the acquisition raw materials and craft production, far-reaching trade routes, and social hierarchisation [86, 90]. However, in the Veneto this strategy ends with this site; assemblages from later Venetian settlements do not show the high-pig pattern, despite demonstrating comparable developments in social stratification and urban settlement organisation, suggesting a strong cultural preference unique to the Etruscan southern plain.

### Divergent patterns of size change

Biometric analyses demonstrated divergent patterns of size change in domestic bovids compared to suids. These trends, in which saw bovids increase in size while pigs decreased, suggest that biometric change was not the result of a universal, external factor like climatic change, but rather the product of anthropic modifications to husbandry practices. Compared to regional diversity in NISP frequencies, size increase was a broad phenomenon which occurred across Northern Italy, even if regions displayed different degrees of change. The precise origin of these increases remains unclear due to the limited data available for the Final Bronze Age and Early Iron Age, but significant transformation in the size of domestic bovids over later prehistory was apparent in the North, South, and Veneto study areas; evaluation of other regions is constrained by sample size. Where it is possible to assess diachronic trends, change in livestock size appeared incremental, developing over the Bronze–Iron Age transition, into the late Iron Age. Metacarpal biometry illustrated size/shape change along the same regression line, conducive with change within a single population. The progressive and pan-regional nature of these trends suggests that they resulted from internal developments common across northern Italian communities rather than the introduction of a new population of livestock, as might follow conquest or colonisation. Body size increase was not limited to urban Etruscan or Venetian areas, nor were large livestock limited to such sites: in contrast to our initial predictions, advances in livestock productivity were therefore independent of urban population density or settlement structures. Broader transformations in animal management must therefore have played a role.

Advances in sheep and cattle husbandry, as indicated by size increase, occurred without similar evidence for an improvement in pig body size. Our results demonstrated a diminution of domestic suids over later prehistory in a continuation of the trend established between the Neolithic and Bronze Age [134]. Previous studies have identified a disconnect in body-size change in domestic cattle and sheep versus pigs during later Italian prehistory [7, 35, 42, 44, 45], but our analysis is the first to show a diachronic decrease in carcass size over this period. Pigs in Italy are thought to have increased in size during the Roman Imperial period [40, 135], although in southern areas diminution may have continued into Roman times [41]. Pig husbandry therefore appears to have been more conservative than sheep or cattle exploitation over late prehistory, probably as a result of different management styles and feeding strategies.

### Implications for agricultural strategies

In antiquity, most pigs were raised in free-ranging herds, kept for at least part of the year in local woodlands to take advantage of mast forage [136]. Such systems were common in the Mediterranean until relatively recently [137, 138], and pigs raised in this manner are dependent on natural foods part of the year. Extensively raised animals have the opportunity to interbreed with wild boar naturally present in the same woodland. In these systems, confinement and habitat fragmentation has a significant impact on access to wild resources and animals, with consequences for carcass size [139]. Because nutrition is a major element in determining animal health with significant impact on animal size and fertility [115, 140–142], increased population density without food supplementation negatively influences body size. Enclosure–even in a relatively large enclosure of 290 ha–produced a reduction in body size of Italian wild boar due to increased competition and reduced food quantity [138]. Fencing or deforestation during prehistory could have produced a similar effect in domestic pigs. Confinement of pig herds did not necessarily equate to keeping animals in stalls, but of limiting their range of movement. If accompanied by a decrease in breeding age [15], such changes could produce significant size diminution. A reduction of interbreeding with phenotypically larger wild boar could also have had a role [134], potentially compounding a size decrease resulting from changes in territorial range and inter-animal competition. The variability and lack of separation between wild and domestic populations in pig histograms suggests greater interbreeding during the Bronze Age than in subsequent periods. Separation of wild and domestic *Sus* through enclosure or a reduction in the number of wild animals through hunting pressure (insinuated by the reduction in wild taxa on archaeological sites, cf. [7]) would reduce gene flow with larger wild pigs. Habitat reduction through deforestation may have contributed to the decline of wild boar, but this appears to have been relatively minor, since pollen analyses from the region do not suggest significant changes in forest cover during the Iron Age [64, 69].

Bovid management strategies are more complicated to reconstruct due to the greater number of products for which they were exploited. A number of factors, alone or in combination, may have provoked an increase in bovid size: e.g. changes to feeding strategy, access to pasture, mobility patterns, or gene flow. Broader changes in agricultural production during the Iron Age suggest greater specialisation (e.g. of viticulture) and surplus output, as an increase in population and non-farming craftsmen would require a concurrent rise in cereal production. In Iron Age Germany, isotopic studies suggest a surplus was achieved through manuring and an expansion of arable cultivation, albeit with a significant degree of local variability [96]; however, similar studies are lacking for northern Italy, and the mechanisms through which greater agricultural output was achieved are open to debate. While an expansion in cultivated area or the exploitation of heavier soils may have encouraged the development of larger cattle for traction, similar pressures would have been present in densely inhabited areas during the Bronze Age. Furthermore, at least in the Veneto, increases in cattle size around the Bronze–Iron Age transition pre-date the diffusion of iron agricultural tools. Body size is, however, associated with milk production [143], an increase in which would have been desirable in both large and small bovids. A change in vertical mobility patterns in Switzerland during the twelfth century BC has been argued to result from greater focus on secondary product exploitation [144]. The impact of this change in strategy on animal biometry was not included in the study. However, the authors highlight the relationship between changes in animal management and concurrent cultural change, which presents interesting parallels for our data. In northern Italy, our earliest evidence for cattle size increase dates to the late Bronze–Iron Age transition–a period of significant cultural change associated with the spread of proto-Villanovan culture during the Final Bronze Age and subsequent development of distinct regional cultures during the Early Iron Age. A thorough understanding of the impact of this development on arable agriculture is lacking, but an intensification of trade and craft specialisation are apparent in the archaeological record [145]. As in Switzerland, cultural change may have prompted a change in management strategy, supported in northern Italy by greater circulation of animals or husbandry knowledge/skills, which moved alongside other materials in better integrated trade networks.

Ultimately, conclusive identification of the cause(s) underlying these developments is not possible at this juncture. However, this regional synthesis sets a basis to further explore the subject with targeted analyses (e.g. isotopes, DNA), as well as to identify the areas and periods that need further research. The results presented here demonstrate that NISP frequencies displayed a high degree of regionality, and the lack of correlation between NISP data and mean body size suggests that changes in animal management were more influential than the focus on a particular taxon. Quantitative assessment of mortality patterns, which are central to reconstruction of husbandry regimes, was beyond the scope of this paper due to the diverse methods used in the literature to record animal age. However, our consideration of metacarpal biometry indicated that changes in sex ratios, which are closely linked to slaughter strategies, were not the primary cause of size improvement. Considering differences in taxon preference and the diverse settlement patterns, material cultures, and languages present across the study areas, it is interesting to see that the overall tendency in livestock size change–bigger cattle and sheep, smaller pigs–occurred across the whole region, although with different rhythms. Large livestock were probably universally desired, but due to various constraints unattainable or too impractical to sustain. Our results suggest that these limitations changed in later prehistory, well before the Roman conquest.

## Conclusion

An increase in livestock size is a key feature of the Romanisation of Western Europe, and one believed to result from changes in farming strategies and the importation of larger Roman livestock. This change was a reversal of millennia of size diminution beginning with domestication, and it signalled significant changes in how animals were managed. However, in northern Italy livestock increased significantly in size prior to the Roman conquest. Etruscan/Roman civilisation was thought to have had a key role in the development of large cattle and sheep, but our results demonstrated an earlier size increase in domestic bovids across northern Italy, regardless of cultural context or degree of urbanisation. This process was visible in all regions with sufficient samples. Metacarpal biometry and the distribution of size data suggest an incremental, internally mediated process. The origin of the increase is difficult to identify due to a lacuna in the data, but it is now clear that the late Bronze Age or Early Iron Age was the starting point. Unlike cattle and sheep, pigs decreased in size over later prehistory, a continuation of the established trend that began in the Neolithic. This diminution implies that herding strategies of domestic suids were significantly different from that of bovids, displaying greater continuity with established trends. This pattern is present even south of the River Po, where zooarchaeological evidence for the species exploited suggests a dramatic shift in husbandry strategy in conjunction with Etruscan culture. In contrast to the widespread trends observed in biometric analysis, the relative abundance livestock taxa varied significantly between study regions. In some instances, husbandry patterns are highly culturally specific, such as the emergence of a pig-dominated pattern on Etruscan sites; in other areas assignment of trends to specific culture was more difficult and may be complicated by local responses to topography. Nevertheless, the marked regionality visible in NISP frequencies did not impact the overall trajectory of body size change, which was observed across the northern Italy.

This evolution of livestock size, across regions with diverse taxon frequencies, material cultures, and settlement patterns, represents a previously undocumented pan-regional improvement in cattle and sheep during later prehistory. It signals a significant change in the management of domestic bovids, in which a shift in the aims of husbandry, agricultural knowledge, feeding strategies, or mobility regimes may have played a role. Future DNA and isotopic analyses will help clarify the role of herd mobility and livestock trade in catalysing these changes. The socio-economic forces and cultural preferences that shaped these patterns also deserve further consideration [146–148], particularly in relation to how social stratification and site catchment areas impacted access to resources like pasture, fodder, and desirable animals. Economic connectivity and complexity were crucial to the expansion of surplus agricultural production and the rise of the Roman market economy [149–152], and our zooarchaeological data provide evidence that agricultural production began to shift across northern Italy well before the Roman époque. Advances in livestock management thus follow a similar trajectory as other forms of archaeological evidence that developed significantly from the Final Bronze Age/Early Iron Age across the first millennium BC: an expansion of trade, wealth consolidation, and organised resource exploitation [57, 148, 149, 152–154]. Agricultural improvements in late prehistoric Italy were not the result protohistoric urbanism or Roman innovation, but part of an earlier incremental phenomenon, likely driven by the same forces that shaped the broader socio-economic landscape of the region during later prehistory.

## Supporting information

S1 Table List of sites with data on chronology, analysis group, NISP, number of LSI values, and associated references. (DOCX)

S2 Table Results of chi-squared tests on livestock NISP. (XLSX)

S3 Table Results of Mann-Whitney U tests on LSI values. (XLSX)
