# Supplementary material for: Pre-Roman improvements to agricultural production: Evidence from livestock husbandry in late prehistoric Italy
Source: PLoS One. 2018 Dec 31;13(12):e0208109. doi: 10.1371/journal.pone.0208109 (PMC6312331; doi:10.1371/journal.pone.0208109)
Supplement: S1 Table — (DOCX) [file pone.0208109.s001.docx]

**S1 Table.
List of sites with data on chronology, analysis group, NISP, number of LSI values, and associated references**

*Cattle, sheep/goat, pig
**Ritual assemblage; excluded from NISP analysis.

| Map id | Site name | Area/study | Period | Sub-period | Study area. NISP period | Biometric period | NISP 3 dom.* | n. LSI values | Refs |
| --- | --- | --- | --- | --- | --- | --- | --- | --- | --- |
| 1 | Amolara |  | Bronze Age | Recent | Veneto.R | BM-R | 690 | 23 | [1, 2] |
| 2 | Baggiovara | Opera Pia Bianchi | Bronze Age | Middle | South.M | BM-R | 438 | 64 | [3] |
| 3 | Bazzano Rocca |  | Bronze Age | Middle | South.M | BM-R | 102 | 5 | [4] |
| 4 | Bologna | Via Ca Selvatica | Iron Age |  | South.IA2 | IA2 | NA | 5 | [5] |
| 5 | Bologna | Via S. Caterina | Iron Age |  | South.IA2 | IA2 | 180 | 9 | [5] |
| 6 | Bologna | Via Foscolo-Frassinago | Iron Age |  | South.IA1 | IA1 | 312 | 4 | [6] |
| 7 | Bovolone |  | Bronze Age | Recent | North.R | BM-R | 2155 | 164 | [1, 2] |
| 8 | Campestrin |  | Bronze Age | Recent | Veneto.R | BM-R | 224 | 24 | [1, 2] |
| 9 | Casale di Rivalta |  | Iron Age |  | South.IA2 | IA2 | 630 | 53 | [7] |
| 10 | Case Vandelli |  | Iron Age |  | South.IA2 | IA2 | 182 | 12 | [5] |
| 11 | Castellaro del Vho | Bronzo medio - Scavi 1995 + 1996-1999 | Bronze Age | Middle | South.M | BM-R | 1929 |  | [8, 9] |
| 12 | Castellaro del Vho | Bronzo recente - Scavi 1995 + 1996-1999 | Bronze Age | Recent | South.R | BM-R | 224 |  | [8, 9] |
| 13 | Castellaro Lagusello |  | Bronze Age | Middle-Recent | North.M-R | BM-R | 3192 | 18 | [10] |
| 14 | Castelrotto |  | Iron Age |  | North.IA2 | IA2 | 2429 | 278 | [11] |
| 15 | Castenaso | Bologna - Castenaso | Iron Age |  | South.IA1 | IA1 | 1278 | 2 | [12] |
| 16 | Cavalzara |  | Bronze Age | Recent-Final | North.R-F | BR-IA0 | 145 | 14 | [13] |
| 17 | Colognola ai Colli |  | Iron Age |  | North.IA2 | IA2 | 1829 | 118 | [14] |
| 18 | Concordia Sagittaria | Quartiere Nord-Ovest 2001 | Iron Age |  | Friuli.IA0 | BR-IA0 | 1100 | 63 | [15, 16] |
| 19 | Feniletto |  | Bronze Age | Recent | North.R | BM-R | 84 | 17 | [17] |
| 20 | Fidenza |  | Iron Age |  | South.IA2 | IA2 | NA | 9 | [5] |
| 21 | Fiorano Modenese |  | Iron Age |  | South.IA1 | IA1 | 107 |  | [18] |
| 22 | Fondo Paviani | de Grossi 2015 | Bronze Age | Recent-Final | Veneto.R-F | BR-IA0 | 174 |  | [19] |
| 23 | Fondo Paviani | Riedel 1979 | Bronze Age | Recent-Final | Veneto.R-F | BR-IA0 | 244 | 41 | [13] |
| 24 | Fondo Paviani | de Grossi 2015 | Bronze Age | Recent | Veneto.R | BM-R | 762 |  | [19] |
| 25 | Forcello | Scarpa + Trentacoste PhD | Iron Age |  | South.IA2 | IA2 | 18446 | 1476 | [20, 21] |
| 26 | Frattesina |  | Iron Age |  | Veneto.IA0 | BR-IA0 | 533 |  | [19] |
| 27 | Frattesina |  | Bronze Age | Final | Veneto.F | BR-IA0 | 1145 |  | [19] |
| 28 | Gaggio | Struttura 1 + capanna alfa | Bronze Age | Middle | South.M | BM-R | 241 | 25 | [22] |
| 29 | Gradisca | Final Bronze Age - Early Iron Age | Bronze Age - Iron Age | Final | Friuli.F-IA0 | BR-IA0 | 76 | 5 | [23] |
| 30 | Gradisca | Iron Age I | Iron Age |  | Friuli.IA1 | IA1 | 437 | 26 | [23] |
| 31 | Gradisca | Iron Age II | Iron Age |  | Friuli.IA2 | IA2 | 564 | 16 | [23] |
| 32 | Gradiscutta | Centes | Iron Age |  | Friuli.IA2 | IA2 | 205 | 6 | [24, 25] |
| 33 | Isolone |  | Bronze Age | Recent | North.R | BM-R | 2925 | 395 | [26] |
| 34 | Larda I |  | Bronze Age | Recent | Veneto.R | BM-R | 525 | 27 | [1, 2] |
| 35 | Larda II |  | Bronze Age | Recent | Veneto.R | BM-R | 446 | 25 | [1, 2] |
| 36 | Lavagnone | Sector A + E | Bronze Age | Middle | North.M | BM-R | 2432 | 228 | [27] |
| 37 | Marzabotto | V 3 | Iron Age |  | South.IA1 | IA1 | 180 | 42 | [5] |
| 38 | Marzabotto | US 27/29 | Iron Age |  | South.IA1 | IA1 | 197 | 13 | [5] |
| 39 | Marzabotto | US 8 | Iron Age |  | South.IA2 | IA2 | 425 | 43 | [5] |
| 40 | Marzabotto | Casa 1 | Iron Age |  | South.IA2 | IA2 | 4783 | 47 | [28] |
| 41 | Mirandola | Arginone | Iron Age |  | South.IA2 | IA2 | 144 | 1 | [29] |
| 42 | Mirandola | Barchessone Cappello | Iron Age |  | South.IA2 | IA2 | 149 | 13 | [30] |
| 43 | Monte Bibele | Ambiente 3/3a/14/24b | Iron Age |  | South.IA2 | IA2 | 1743 | 35 | [31, 32] |
| 44 | Monte Castellaccio |  | Bronze Age | Middle | Romagna.M | BM-R | 405 |  | [33] |
| 45 | Monte Leoni |  | Bronze Age | Middle | South.M | BM-R | 494 |  | [34, 35] |
| 46 | Monte Zoppega |  | Bronze Age - Iron Age |  | North.IA0 | BR-IA0 | 182 | 12 | [13] |
| 47 | Montebello |  | Bronze Age | Recent-Final | North.R-F | BR-IA0 | 184 |  | [36] |
| 48 | Muraiola |  | Bronze Age | Middle | North.M | BM-R | 4185 | 36 | [37] |
| 49 | Noceto** | Vasca lignea | Bronze Age | Middle | South.M | BM-R | 609 | 101 | [38] |
| 50 | Nogarole Rocca | I camponi | Bronze Age | Middle | North.M | BM-R | 998 | 71 | [39] |
| 52 | Oderzo | via dei Mosaici - A (IX-VIII) | Iron Age |  | Veneto.IA0 | BR-IA0 | 26 | 2 | [40] |
| 53 | Oderzo | via dei Mosaici - E+G (IV-II) | Iron Age |  | Veneto.IA1 | IA1 | 54 | 4 | [40] |
| 54 | Oderzo** | Casa preromana Settore S-E | Iron Age |  | Veneto.IA2 | IA2 | 7 | 2 | [41] |
| 55 | Padova | Largo Europa - area 1000 | Iron Age |  | Veneto.IA2 | IA2 | 172 |  | [42] |
| 56 | Padova | Via Dietro Duomo | Iron Age |  | Veneto.IA2 | IA2 | 174 | 8 | [43] |
| 57 | Peschiera |  | Bronze Age | Middle-Recent | North.M-R | BM-R | 385 | 37 | [44] |
| 58 | Pilastri Bondeno | Sondaggi 1989 | Bronze Age | Middle-Recent | South.M-R | BM-R | 157 | 18 | [45] |
| 59 | Poggio Rusco |  | Bronze Age | Middle | South.M | BM-R | 132 | 7 | [46] |
| 60 | Poviglio | Villaggio grande - MBA | Bronze Age | Middle-Recent | South.M-R | BM-R | 797 |  | [47] |
| 61 | Poviglio | Villaggio grande – RBA | Bronze Age | Recent | South.R | BM-R | 1411 |  | [47] |
| 62 | Poviglio | Villaggio piccolo – MBA | Bronze Age | Middle | South.M | BM-R | 1922 | 131 | [48, 49] |
| 63 | Pozzuolo | Braida Roggia - Bronze Age | Bronze Age | Recent | Friuli.R | BM-R | 338 | 3 | [50, 51] |
| 64 | Pozzuolo | Braida Roggia - Iron Age | Iron Age |  | Friuli.IA0 | BR-IA0 | 36 | 9 | [50] |
| 65 | Pozzuolo | Excavations 1980-83 | Iron Age |  | Friuli.F-IA0 | BR-IA0 | 2021 | 168 | [52, 53] |
| 66 | Quarto del Tormine | Mozzecane | Bronze Age | Middle | North.M | BM-R | 257 | 14 | [54] |
| 67 | Riccione | IperCoop | Bronze Age | Middle-Recent | Romagna.M-R | BM-R | 83 | 2 | [55] |
| 68 | Rubiera | Ca del Cristo | Iron Age |  | South.IA2 | IA2 | 43 | 8 | [56] |
| 69 | Sabbionara di Veronella |  | Bronze Age | Recent | North.R | BM-R | 1046 |  | [57] |
| 70 | San Claudio |  | Iron Age |  | South.IA1 | IA1 | 248 | 6 | [58] |
| 71 | San Giorgio di Valpolicella |  | Iron Age |  | North.IA2 | IA2 | 665 |  | [59] |
| 72 | San Giuliano |  | Bronze Age | Middle-Recent | Romagna.M-R | BM-R | 86 | 72 | [60] |
| 73 | Santorso |  | Iron Age |  | North.IA2 | IA2 | 1837 | 116 | [61] |
| 74 | Solarolo | via Odriere Settore 1 | Bronze Age | Middle | Romagna.M | BM-R | 2111 | 112 | [62] |
| 75 | Spina | Excavations 2007-2009 | Iron Age |  | South.IA2 | IA2 | 300 | 37 | [63] |
| 76 | Spina |  | Iron Age |  | South.IA2 | IA2 | 5100 |  | [64] |
| 77 | Tabina di Margreta | Etruscan | Iron Age |  | South.IA2 | IA2 | 72 |  | [65] |
| 78 | Tabina di Margreta | Bronze Age | Bronze Age | Middle | South.M | BM-R | 520 | 48 | [66] |
| 79 | Terranegra | Depellegrin & Tecchiati - BR | Bronze Age | Recent | Veneto.R | BM-R | 940 | 97 | [67, 68] |
| 80 | Terranegra | Depellegrin & Tecchiati - IA | Iron Age |  | Veneto.IA1 | IA1 | 1606 | 180 | [67, 68] |
| 81 | Tombola |  | Bronze Age | Middle | Veneto.M | BM-R | 1017 | 81 | [1, 2] |
| 82 | Treviso | Piazza S. Andrea | Iron Age |  | Veneto.IA2 | IA2 | 222 |  | [69] |
| 83 | Vallette |  | Bronze Age | Middle | Veneto.M | BM-R | 215 | 32 | [1, 2] |
| 84 | Verucchio | Scavo 1963 | Iron Age |  | Romagna.IA1 | IA1 | 619 | 99 | [70] |

**References**

1. Bertolini M. Allevamento e lavorazione della materia dura animale nell’età del Bronzo nella pianura veneta sudoccidentale. Ph.D. thesis, Università degli Studi di Ferrara; 2014. Availble from: http://eprints.unife.it/895/.

2. Bertolini M, Zanini S, Thun Hohenstein U. Nuovi dati sullo sfruttamento e gestione delle risorse animali tra il Bronzo antico ed il Bronzo recente nei territori del medio-basso Veronese e il basso Polesine. In: Leonardi G, Tiné V, editors. Preistoria e protostoria del Veneto. Firenze: Istituto Italiano di Preistoria e Protostoria; 2015. p. 321–6. (Studi di preistoria e protostoria; 2).

3. De Grossi Mazzorin J, Epifani I. L'analisi dei resti faunistici provenienti dalla terramara di Baggiovara (MO). In: De Grossi Mazzorin J, Curci A, Giacobini G, editors. Economia e ambiente nell'Italia padana nell'età del Bronzo: le indagini bioarcheologiche. Bari: Edipuglia; 2013. p. 223–33. (Beni archeologici - conoscenza e tecnologie quaderno; 11).

4. Curci A, Maini E. La fauna dell’età del Bronzo della Rocca di Bazzano. In: Burgio R, Campagnari S, editors. Il Museo Civico Archeologico “Arsenio Crespellani” nella Rocca dei Bentivoglio di Bazzano. Bazzano: Museo civico "Arsenio Crespellani"; 2008. p. 47–54.

5. Farello P. L'Emilia dal VI e V secolo a.C.: caccia e allevamento. Atti del I° convegno nazionale di archeozoologia, Rovigo 5–7 marzo 1993. Rovigo: Centro Polesano di Studi Storici, Archeologici ed Etnografici; 1995. p. 209–34. (Padusa Quaderni; 1).

6. Farello P. I reperti ossei. In: Ortalli J, Pini L, editors. Lo scavo archeologico di Via Foscolo-Frassinago a Bologna: aspetti insediativi e cultura materiale. Firenze: All'Insegna del Giglio; 2002. p. 93–104. (Quaderni di archeologia dell’Emilia-Romagna; 7).

7. Farello P. Casale di Rivalta. Reperti faunistici. In: Ambrosetti G, Macellari R, Malnati L, editors. Vestigia crustunei, insediamenti etruschi lungo il corso del Crostolo. Reggio Emilia: Edizioni Tecnostampa; 1990. p. 214–56. (Archaeologica regiensia; 5).

8. Di Martino S. GA, Di Giancamillo (2001) La fauna. In: Frontini P, editor. Castellaro del Vhò Campagne di scavo 1996-1999: scavi delle civiche raccolte archeologiche di Milano. Milano: Comune di Milano, Settore cultura e spettacolo, raccolte archeologiche e numismatiche; 2001. p. 203–14.

9. di Martino S. I resti faunistici. In: Frontini P, editor. Castellaro del Vhò Campagna di scavo 1995 Scavi delle civiche raccolte archeologiche di Milano. Milano: Milano, Comune di Milano, Settore cultura, musei e mostre; 1997. p. 159–72.

10. Malerba G, Rocci Ris A, Giacobini G, Fasani L. I macromammiferi del sito dell’età del Bronzo di Castellaro Lagusello (Monzambano – MN): i primi dati. In: Malerba G, Visentini P, editors. Atti del 4° convegno nazionale di archeozoologia (Pordenone, 13-15 novembre 2003). Pordenone: Comune di Pordenone, Museo Archeologico; 2005. p. 223–35. (Quaderni del Museo Archeologico del Friuli Occidentale; 6).

11. Riedel A. The fauna of the Iron Age site of Castelrotto (Verona). Padusa. 1985;21:55–98.

12. Farello P. L'Insediamento di Castenaso: allevamento, caccia e pesca in un sito Villanoviano. In: Forte M, von Elesvon P, editors. La pianura bolognese nel Villanoviano: insediamenti della prima età del Ferro. Firenze: All'Insegna del Giglio; 1994. p. 218–23.

13. Riedel A. La fauna di alcuni insediamenti preistorici del territorio veronese. Atti del Museo Civico di Storia Naturale di Trieste. 1979;31(1):41–73.

14. Riedel A. The fauna of the excavations of Colognola ai Colli. Bollettino del Museo Civico di Storia Naturale di Verona. 1984;11:277–318.

15. Tagliacozzo A, Cerilli D, Albertini D. Concordia Sagittaria - analisi dei resti ossei, note preliminari. In: La protostoria tra Sile e Tagliamento: antiche genti tra Veneto e Friuli. Padova: Esedra editrice; 1996. p. 290–7.

16. Pino Uria B, Tagliacozzo A. Studio archeozoologico dei livelli protostorici del quartiere nord-ovest di Concordia Sagittaria (Venezia) nel quadro delle faune dell’Italia nord-orientale. Quaderni di Archeologia del Veneto. 2001;17:141–57.

17. Riedel A. Die Fauna von Feniletto (Verona). Rivista di Archeologia. 1982;6:28–30.

18. Farello P. Fiorano Modenese. Reperti faunistici. In: Ambrosetti G, Macellari R, Malnati. L, editors. Rubiera, "principi" etruschi in Val di Secchia. Reggio Emilia: Comune di Reggio Emilia; 1989. p. 179–84. (Archeologia regiensia; 4).

19. De Grossi Mazzorin J. Fondo Paviani e Frattesina: economia animale di due *central places* della tarda età del Bronzo veneta. In: Leonardi G, Tiné V, editors. Preistoria e protostoria del Veneto. Firenze: Istituto Italiano di Preistoria e Protostoria; 2015. p. 389–400. (Studi di preistoria e protostoria; 2).

20. Scarpa G. La fauna. In: de Marinis RC, editor. Gli Etruschi a nord del Po. Vol. 1. Udine: Companotto Editore; 1988. p. 184–92.

21. Trentacoste A. The Etruscans and their animals: the zooarchaeology of Forcello di Bagnolo San Vito (Mantova). Ph.D. thesis, University of Sheffield; 2014. Available from: http://etheses.whiterose.ac.uk/6553/.

22. De Grossi Mazzorin J, Minniti C, Saracino F. L'analisi dei resti faunistici provenienti dalla struttura 1 della terramara di Gaggio (MO). In: De Grossi Mazzorin J, Curci A, Giacobini G, editors. Economia e ambiente nell'Italia padana nell'età del Bronzo: le indagini bioarcheologiche. Bari: Edipuglia; 2013. p. 247–55. (Beni archeologici - conoscenza e tecnologie; 11).

23. Petrucci G. La fauna. In: Càssola Guida P, Balista C, editors. Gradisca di Spilimbergo: indagini di scavo in un castelliere protostorico 1987–1992. Roma: Edizioni Quasar; 2007. p. 367–405. (Studi e ricerche di protostoria mediterranea; 7).

24. Riedel A, Tecchiati U. I resti faunsitici. Aquileia Nostra. 2003;74:714–8.

25. Riedel A, Tasca G, Tecchiati U. I resti faunistici del V sec. a.C. di Centes di Gradiscutta di Varmo (UD). In: Malerba G, Visentini P, editors. Atti del 4° convegno nazionale di archeozoologia (Pordenone, 13-15 novembre 2003). Pordenone2006. p. 293–300. (Quaderni del Museo Archeologico del Friuli Occidentale; 6).

26. Riedel A. La fauna del villaggio preistorico di Isolone della Prevaldesca. Bollettino del Museo Civico di Storia Naturale - Verona. 1975;2:355–414.

27. De Grossi Mazzorin J, Solinas AM. L'analisi dei resti faunistici provenienti dai settori A ed E della palafitta del Lavagnone. In: De Grossi Mazzorin J, Curci A, Giacobini G, editors. Economia e ambiente nell'Italia padana nell'età del Bronzo: le indagini bioarcheologiche. Bari: Edipuglia; 2013. p. 21–102. (Beni archeologici - conoscenza e tecnologie; 11).

28. Curci A. I dati archeozoologici. In: Govi E, Sassatelli G, editors. Marzabotto La casa della regio IV - insula 2. Vol 2, I materiali. Bologna: Ante Quem; 2010. p. 397–420.

29. Farello P. Mirandola, loc. Arginone. Reperti faunsitici. In: Calzolari M, Malnati L, editors. Gli Etruschi nella Bassa Modenese: nuove scoperte e prospettive di ricerca in un settore dell’Etruria padana. San Felice sul Panaro: Gruppo Studi Bassa Modenese; 1992. p. 273–83.

30. Farello P. Mirandola, loc. Barchessone Cappello, scavo 1991. Impianto produttico di eta' etrusca (IV sec. a.C.). Reperti faunsitici. Quaderni della Bassa Modenese. 1993;24:115–22.

31. Curci A, Baldassarri P, Barone L, Corli S, Fait M, Ferrari V, et al. Lo sfruttamento delle risorse animali dell'abitato di Monte Bibele: la casa 14. Ocnus. 2000;8:93–103.

32. Curci A, Penzo A, Cattabriga S. Animali a Monte Bibele: sacrifici per gli dei, cibo per gli uomini. In: Curci A, Vitali D, editors. Animali tra uomini e dei: archeozoologia del mondo preromano. Bologna: Ante Quem; 2006. p. 111–25. (Studi e scavi, nueva serie; 14).

33. de Grossi Mazzorin J. Archeozoologia delle “ossa di bruti” provenienti dagli scavi della stazione preistorica sul Monte Castellaccio presso Imola. In: Pacciarelli M, editor. La collezione Scarabelli. Vol. 2, Preistoria. Casalecchio di Reno (Bologna): Grafis; 1996. p. 181–218.

34. Bonardi S, Scarpa G. Ricerca paleoecologica a Monte Leoni in Val Parma. Analisi del materiale osteologico. Preistoria Alpina. 1982;18:209–15.

35. Ammerman A, Butler J, Diamond G, Menozzi P, Pals J, Sevink J, et al. Rapporto sugli scavi a Monte Leoni: un insediamento dell'età del Bronzo in Val Parma. Preistoria Alpina. 1976;12:127–54.

36. Balista C, De Guio A, Leonardi G, Ruta Serafini A. La frequentazione protostorica del territorio vicentino: metodologia analitica ed elementi preliminari di lettura interpretativa. Dialoghi di Archaeologia. 1982;2(4):113–36.

37. Riedel A. La Fauna di Muraiola. In: Belemmi L, Salzani L, Squaranti G, editors. Povegliano: l'abitato dell'età del Bronzo della Muraiola. Povegliano: Associazione Balladoro; 1997. p. 77–113.

38. De Grossi Mazzorin J, Saracino F. Analisi archeozoologica della fauna della vasca legnea di Noceto (PR). In: De Grossi Mazzorin J, Curci A, Giacobini G, editors. Economia e ambiente nell'Italia padana nell'età del Bronzo: le indagini bioarcheologiche. Bari: Edipuglia; 2013. p. 189–206. (Beni archeologici - conoscenza e tecnologie; 11).

39. Riedel A. The Bronze Age animal bone deposit of Nogarole Rocca I Camponi (Verona). Padusa. 1992;28:87–104.

40. Tagliacozzo A, Fiore I. Oderzo, via dei Mosaici. I resti faunistici. Quaderni di Archeologia del Veneto. 1992;8:166–73.

41. Tagliacozzo A. Analisi dei resti ossei dell'area di fondazione di una casa nell'area del foro di Oderzo. In: La protostoria tra Sile e Tagliamento: antiche genti tra Veneto e Friuli. Padova: Esedra editrice; 1996. p. 162–3.

42. Tagliacozzo A. Le ossa animali. In: Balista C, Ruta Serafina A, editors. Saggio stratigrafico preso il muro romano di Largo Europa a Padova: nota preliminare. Quarderni di Archeologia del Veneto 91993. p. 95–111.

43. Tagliacozzo A, Cassoli PF. I resti ossei faunistici dell'abitato paleoveneto di Padova, via dietro Duomo. Bollettino del Museo di Padova. 1990;79:111–333.

44. Riedel A. Dei Fauna einer bronzezeitlichen Siedlung bei Peschiera am Gardasee. Rivista di Archeologia. 1982;6:23–7.

45. Farello P. Fauna dell'età del Bronzo dal sito di Pilastri. In: Desantis P, Steffè G, editors. L'insediamento terramaricolo di Pilastri (Bodeno-Ferrara): Prime fasi di una ricerca. Firenze: All'Insegna del Giglio; 1995. p. 98–104.

46. Catalani P. Poggio Rusco (MN): la fauna. Preistoria Alpina. 1984;20:203–10.

47. Riedel A. L'economia animale. In: Bernabò Brea M, Cremaschi M, editors. La terramara di Poviglio: le campagne di scavo 1985–1989. Reggio Emilia: Coopsette; 1989. p. 37–8.

48. Riedel A. La fauna. In: Bernabò Brea M, Cremaschi M, editors. Il Villaggio Piccolo della terramara di S Rosa di Poviglio, Scavi 1987–1992. Firenze: Istituto Italiano di Preistoria e Protostoria; 2004. p. 743–77.

49. Girod A. Malacofauna. In: Bernabò Brea M, Cremaschi M, editors. Il Villaggio Piccolo della terramara di S Rosa di Poviglio, Scavi 1987–1992. Firenze: Istituto Italiano di Preistoria e Protostoria; 2004. p. 779–84.

50. Riedel A. La fauna di Braida Roggia a Pozzuolo del Friuli. Atti del Museo Civico di Storia Naturale di Trieste. 1981;12(1):121–31.

51. Petrucci G. Appendice II: la fauna. In: Càssola Guida P, Borgna E, editors. Pozzuolo del Friuli - 1: i resti della tarda età del Bronzo in località Braida Roggia. Roma: Quasar; 1994. p. 119–227. (Studi e ricerche di protostoria mediterranea; 2)

52. Riedel A. The fauna of the excavation of Pozzuolo del Friuli (1980–1983). Atti del Museo Civico di Storia Naturale do Trieste. 1984;14:215–76.

53. Petrucci G. Pozzuolo del Friuli: i dati dell'archeozoologia. In: Càssola Guida P, Pettarin S, Petrucci G, Giumlia-Mair A, editors. Pozzuolo del Friuli - II, 2: la prima età del Ferro nel settore meridionale del castelliere. Le attività produttive e i resti faunistici. Roma: Quasar; 1998. p. 127–38. (Studi e ricerche di protostoria mediterranea; 5).

54. Riedel A. Mozzecane, Quarto del Tormine. La fauna. Quaderni di Archeologia del Veneto. 1987;3:117–8.

55. Maini E. Le analisi archeozoologiche nel sito di Riccione - Ipercoop. In: De Grossi Mazzorin J, Curci A, Giacobini G, editors. Economia e ambiente nell'Italia padana nell'età del Bronzo: le indagini bioarcheologiche.. Bari: Edipuglia; 2013. p. 317–27. (Beni archeologici - conoscenza e tecnologie; 11).

56. Farello P. Rubiera, Ca' del Cristo. Reperti faunsitici. In: Ambrosetti G, Macellari R, Malnati. L, editors. Rubiera, "principi" etruschi in Val di Secchia. Reggio Emilia: Comune di Reggio Emilia; 1989. p. 139–42. (Archeologia regiensia; 4).

57. Riedel A. La fauna di Sabbionara di Veronella (Verona). In: Salzani L, editor. L’abitato e la necropoli di Sabbionara di Veronella. Cologna Veneta: Editrice Ambrosini; 1993. p. 79–92.

58. Farello P. S. Claudio. Reperti faunistici. In: Ambrosetti G, Macellari R, Malnati L, editors. Vestigia Crustunei. Insediamenti etruschi lungo il corso del Crostolo. Reggio Emilia: Tecnostampa; 1990. p. 133–40. (Archaeologica Regiensia; 5).

59. Riedel A. Considerazioni sulla fauna dell'età del Ferro. In: Salzani L, editor. San Giorgio di Valpolicella. Verona: Banca popolare di Verona; 1992. p. 81–7.

60. de Grossi Mazzorin J. Analisi dei resti faunistici dell'insediamento protostorico di San Giuliano di Toscanella. In: Pacciarelli M, editor. La collezione Scarabelli. Vol. 2, Preistoria. Casalecchio di Reno (Bologna): Grafis; 1996. p. 308–12.

61. Cassoli PF, Tagliacozzo A. La fauna degli scavi 1983-1986 a Santorso, Vicenza (età del Ferro). Preistoria Alpina. 1990;25:165–216.

62. Maini E, Curci A. Le analisi archeozoologiche nel sito di Solarolo - via Ordiere. In: De Grossi Mazzorin J, Curci A, Giacobini G, editors. Economia e ambiente nell'Italia padana nell'età del Bronzo: le indagini bioarcheologiche. Bari: Edipuglia; 2013. p. 295–311. (Beni archeologici - conoscenza e Tecnologie Quaderno; 11).

63. Briccola N, Bertolini M, Thun Hohenstein U. Gestione e sfruttamento delle risorse animali nell’abitato di Spina: analisi archeozoologica dei reperti faunistici. In: Cassai CC, Giannini S, Malnati L, editors. Spina: scavi nell'abitato della città etrusca, 2007-2009. Firenze: Cooperativa Archeologica; 2013. p. 178–87.

64. Riedel A. Notizie preliminari sullo studio della fauna di Spina. Atti dell’Accademia delle Scienze di Ferrara. 1978;(55):27–33.

65. de Grossi Mazzorin J. Breve nota sui resti faunsitici dell'insediamento dell'età del Ferro di Tabina di Magreta. Appendix to Cattani M, Lo scavo di Tabina di Magreta (cave di via Tampellini) e le trace di divisioni agrarie di età etrusca nel territorio di Modena. Quaderni del Museo Civico Archeologico-Etnologico di Modena: Studi di Preistoria e Protostoria. 1994;1:206.

66. de Grossi Mazzorin J. Tabina di Magreta: la terramara e i resti di età etrusca (campagne di scavo 1985–1986). Nota preliminare sulla fauna dell'insediamento della media età di bronzo. In: Modena dalle origini all'anno mille. Modena: Panini; 1988. p. 225–9. (Studi di archeologia e storia; 1).

67. Depellegrin V, Tecchiati U. I resti faunistici dell'abitato protostorico di Terranegra (Legnago, Verona). Annali dell'Università degli Studi di Ferrara. 2016;12(1). Available from: http://annali.unife.it/museologia/article/view/1303.

68. Depellegrin V, Tecchiati U, Salzani L. Die bronze-und eisenzeitlichen Tierknochenfunde von Terranegra (Legnago, Verona). Annalen des Naturhistorischen Museum Wien Serie A für Mineralogie und Petrographie, Geologie und Paläontologie, Anthropologie und Prähistorie. 2018;120:177–94.

69. Tecchiati U, Marconi S, Bianchin Citton E. La fauna protostorica di Treviso alla luce dei dati provenienti dai siti di Piazza Pio X e di Piazza S. Andrea (Palazzo Azzoni Avogadro). Annali dell'Università degli Studi di Ferrara. 2016;12(1). Available from: http://annali.unife.it/museologia/article/view/1323.

70. Farello P. Reperti faunistici da Verucchio (RN) - scavo 1963. Archeologia dell'Emilia-Romagna. 1997;1(1):47–9.
